# Comparative Hg(II) Adsorption on Coffee Husk and Sugarcane Bagasse Biochars: Structure–Function Relationships

**DOI:** 10.3390/molecules31213914

**Published:** 2026-10-30

**Authors:** Jairo F. Gomez-Rojas, César Jaramillo-Páez, Oscar A. Gamba, Jorge L. Gallego, Lis Manrique-Losada

**Affiliations:** 1 Grupo de Investigación de Materiales, Ambiente y Desarrollo (MADE), Programa de Química, Facultad de Ciencias Básicas, Universidad de la Amazonia, Florencia 180002, Colombia; jai.gomez@udla.edu.co; 2 Grupo de Investigación en Química Aplicada a Procesos Ecológicos (QUAPE-UT), Facultad de Ciencias, Universidad del Tolima, Ibagué 730006, Colombia; cajaramillopa@ut.edu.co; 3 Centro de Investigaciones en Catálisis (CICAT), Parque Tecnológico Guatiguará, Universidad Industrial de Santander, km 2 vía el Refugio, Piedecuesta 681011, Colombia; oagamvas@uis.edu.co; 4 Biodiversity, Biotechnology and Bioengineering Research Group—GRINBIO, Department of Engineering, University of Medellin, Medellín 050026, Colombia; jlgallego@udemedellin.edu.co

**Keywords:** biochar, mercury, adsorption mechanism, surface functional groups, toxicity

## Abstract

Mercury contamination in water poses a significant environmental and public health concern because it is toxic, persistent, mobile, and bioaccumulative. This study investigated biochars derived from the perisperm (silver skin) fraction of coffee husk (CH) and sugarcane bagasse (SB) as sustainable adsorbents for Hg(II) removal from water. The biochars were produced at 800 °C, characterized by N_2_ adsorption–desorption measurements at approximately −198 °C, SEM–EDS, XRD, XRF, FTIR, and XPS, and evaluated through kinetic, equilibrium, and thermodynamic studies of Hg(II) adsorption. SB exhibited a higher BET surface area (599 vs. 525 m^2^ g^−1^), larger pore volume (0.298 vs. 0.216 cm^3^ g^−1^), and lower micropore fraction (63.4 vs. 86.3%) than CH. These textural differences were consistent with the faster Hg(II) adsorption observed for SB. The Avrami and Elovich models best described the adsorption kinetics (R^2^ = 0.980–0.996). At the same time, Freundlich and Langmuir adequately described the equilibrium data; their relative performance depended on biochar type and temperature. The Langmuir-estimated maximum adsorption capacity (q_m_) increased with temperature, reaching 45.05 mg g^−1^ for SB and 45.25 mg g^−1^ for CH at 318.15 K. The thermodynamic analysis yielded negative enthalpy and Gibbs free-energy changes for both biochars, with ΔH° values of −71.4 and −52.8 kJ mol^−1^ and ΔG°_(298)_ values of −39.3 and −37.6 kJ mol^−1^ for SB and CH, respectively. FTIR and XPS supported the involvement of oxygen-containing surface environments in Hg(II) retention.

## 1. Introduction

Mercury contamination in aquatic systems remains a critical global environmental and public health concern because of its persistence, mobility, toxicity, and ability to bioaccumulate and biomagnify through food webs [1,2]. Mercury is released from natural processes, including volcanic activity, weathering of mercury-bearing rocks, and forest fires; however, anthropogenic inputs particularly artisanal and small-scale gold mining, coal combustion, non-ferrous metal smelting, cement production, waste incineration, and industrial effluents have substantially increased its environmental circulation [3,4,5]. After atmospheric transport and deposition or direct discharge, mercury reaches rivers, lakes, groundwater, and sediments, where it can exist as elemental mercury (Hg(0)), inorganic species predominantly Hg(II) and organic compounds, especially methylmercury (MeHg). Its speciation, mobility, and bioavailability depend on pH, redox conditions, salinity, and interactions with sulfide, dissolved organic matter, and suspended particles. Inorganic Hg(II) is particularly relevant because it is relatively soluble, can bind strongly to organic and mineral surfaces, and may be converted by anaerobic microorganisms in sediments into MeHg, its most bioavailable and toxic form [6,7,8].

Various treatment technologies have been developed for Hg(II) removal from aqueous systems, including chemical precipitation [9], membrane filtration [10], and electrocoagulation [11]. While these methods can achieve high removal efficiencies, many suffer from limitations such as high operational costs, sludge generation, membrane fouling, and energy demand, which limit their large-scale. In this context, adsorption has emerged as one of the most promising alternatives due to its operational simplicity, effectiveness at low concentrations, and compatibility with low-cost materials [12].

Among emerging adsorbents, biochar has gained significant attention as a sustainable material produced from biomass residues through pyrolysis under oxygen-limited conditions. Its physicochemical properties, including surface functional groups, pore structure, and mineral content, can be tailored through precursor selection and thermal treatment, enabling the removal of a wide range of contaminants [5,13,14]. The adsorption of Hg(II) onto biochar is a complex process involving multiple mechanisms such as surface complexation, ion exchange, electrostatic interactions, precipitation, and pore diffusion [15,16]. However, the relative contribution of these mechanisms strongly depends on the characteristics of the precursor and the resulting biochar structure.

Agroindustrial residues have emerged as attractive precursors for biochar production due to their abundance, low-cost, and offer potential for waste valorization. In particular, lignocellulosic residues derived from agricultural processing industries offer significant advantages because they can be converted into porous carbon materials while simultaneously addressing waste management challenges [17]. Recent advances in biomass-derived sorbents include NaOH-treated spent coffee grounds for methylene blue adsorption [18] and vertically aligned alginate aerogels for solar-enhanced uranium extraction from seawater [19], illustrating the potential of chemical modification and structural engineering to improve adsorption performance. However, these approaches involve additional preparation steps, motivating the investigation of non-activated biochars whose adsorption performance can be assessed in relation to their precursor-derived properties.

Coffee-processing residues have attracted increasing interest as low-cost and sustainable adsorbents due to their lignocellulosic composition, carbon content, and surface functional groups, which can contribute to contaminant retention in aqueous systems [20,21,22]. Different residues, including coffee pulp, coffee husks, spent coffee grounds, and coffee parchment, have been evaluated either as untreated biosorbents or after conversion into biochar. Of particular relevance to mercury remediation, Mora Alvarez et al. (2018) reported 97% Hg(II) removal using untreated exhausted coffee waste, with a maximum adsorption capacity of 31.75 mg g^−1^, highlighting the potential of coffee-derived materials for mercury retention [23]. Paredes-Laverde et al. (2018) demonstrated the applicability of untreated coffee husks for norfloxacin removal [24], while Guimarães et al. (2020) evaluated coffee husk-derived biochars for Fe(II) adsorption, reporting maximum capacities of approximately 110 mg g^−1^ [25]. Similarly, Gora et al. (2022) investigated spent coffee grounds for Cu(II), Pb(II), Zn(II), and Ni(II) removal [26], whereas Carnier et al. (2022) evaluated biochars derived from spent coffee grounds and coffee parchment for Cd(II) and Pb(II) adsorption [27]. More recently, Hu et al. (2025) demonstrated that KOH modification of spent-coffee-ground biochar enhanced its adsorption performance toward Cu(II), Pb(II), Cd(II), and Cr(III) [28]. These studies demonstrate the potential of different coffee-processing residues for contaminant removal and the influence of precursor selection and material modification on adsorption performance. Nevertheless, the Hg(II) adsorption behavior of unmodified coffee husk-derived biochar remains insufficiently explored, particularly regarding the combined contributions of pore architecture, surface chemistry, and mineral composition to mercury retention.

Sugarcane bagasse has attracted considerable interest as a low-cost precursor for biochar production due to its high lignocellulosic content, widespread availability, and potential for conversion into carbonaceous adsorbents [29]. Pyrolysis modifies its pore structure, surface chemistry, and mineral composition, thereby influencing its adsorption performance toward potentially toxic metals [30,31]. Tehreem et al. (2022) demonstrated the applicability of sugarcane bagasse biochar for Pb(II), Ni(II), and Cu(II) removal [32], while Inyang et al. (2011) reported that biochar derived from anaerobically digested bagasse exhibited substantially higher Pb(II) adsorption capacity than biochar produced from raw bagasse, highlighting the influence of precursor composition and mineral-associated adsorption mechanisms [33]. Other studies have similarly associated metal retention by sugarcane-derived biochars with differences in ash content, surface area, and sulfur-containing components [34]. Of particular relevance to mercury remediation, Giraldo et al. (2020) reported Hg(II) removal efficiencies above 90% using thermally treated and chemically activated sugarcane bagasse-derived adsorbents, associating mercury retention with oxygen-containing surface functionalities and aromatic carbon structures [35]. Homagai et al. (2022) further demonstrated the Hg(II) adsorption potential of charred sugarcane bagasse, reporting a maximum adsorption capacity of 125 mg g^−1^ for the unmodified material, which increased to 333.34 mg g^−1^ after chemical xanthation [36]. These findings demonstrate the potential of sugarcane bagasse-derived biochars for heavy-metal removal and highlight the influence of precursor characteristics, mineral composition, and surface modification on adsorption performance [37]. However, the combined contributions of pore architecture, carbonaceous functional groups, and mineral-associated environments to Hg(II) retention by unmodified sugarcane bagasse biochar require further investigation.

Despite the growing body of literature, important knowledge gaps therefore remain. First, many studies reporting high Hg(II) adsorption capacities rely on chemically activated, functionalized, or composite biochars, making it difficult to distinguish the intrinsic contribution of precursor-derived surface chemistry, mineral phases, and pore structure. Second, coffee-derived residues and sugarcane bagasse are generally investigated separately and under different pyrolysis and adsorption conditions, preventing direct comparison of their intrinsic Hg(II) adsorption behavior. Third, the relationship between precursor composition, mineralogy, pore structure, surface functionality, and Hg(II) uptake remains insufficiently understood for non-activated biochars produced under equivalent thermal conditions. This limitation is particularly relevant for agroindustrial residues with contrasting inorganic and lignocellulosic compositions, since precursor-derived mineral phases and surface functionalities may promote fundamentally different Hg(II) retention pathways.

Coffee silverskin and sugarcane bagasse were selected to explore the valorization of residues associated with coffee and sugarcane processing, two agricultural value chains relevant to Caquetá in the Colombian Amazon [38]. Beyond this regional relevance, precursor composition can influence the surface area and surface functionality developed during pyrolysis [39,40], while experimental evidence for bagasse-derived and wood-derived biochars shows that differences in surface chemical environments can lead to distinct Hg(II) retention mechanisms [16], providing a rationale for comparing both residues under identical thermal conditions.

Therefore, this study aims to compare Hg(II) adsorption by two non-activated biochars, coffee husk perisperm (silver skin) biochar (CH) and sugarcane bagasse biochar (SB), both produced by direct pyrolysis at 800 °C under oxygen-restricted conditions. A comprehensive characterization approach combined with kinetic and equilibrium analyses was employed to elucidate the structure–adsorption relationships governing Hg(II) uptake. By producing both biochars under identical thermal conditions and without chemical activation or post-pyrolysis functionalization, this work seeks to isolate the influence of precursor-derived properties on Hg(II) adsorption and to determine how differences in surface chemistry, mineral composition, and pore structure govern mercury retention pathways. This comparative approach provides a basis for assessing the intrinsic potential of these agroindustrial residues as sustainable and low-cost adsorbents for mercury-contaminated water.

## 2. Results and Discussion

### 2.1. Textural Evolution and Micropore Development Derived from BET Analysis

The N_2_ adsorption–desorption isotherms and pore size distributions of CH and SB are presented in Figure 1. According to the IUPAC classification, both materials exhibit type IV(a) adsorption–desorption isotherms with H4-type hysteresis loops, characteristic of micro–mesoporous carbonaceous materials containing a significant contribution of narrow slit-shaped pores [41]. The sharp adsorption observed at low relative pressures (P/P_0_ < 0.1) indicates extensive micropore filling, whereas the hysteresis loop appearing at intermediate relative pressures reveals the presence of narrow mesopores and pore network effects [42].

The Type IV(a) isotherm and the observed hysteresis indicate the presence of mesoporosity associated with capillary condensation, while the pronounced N_2_ uptake at low relative pressures suggests an additional contribution from micropore filling [41]. Such combined micro–mesoporous characteristics have also been reported for coffee-derived biochars produced at high pyrolysis temperatures [41]. The BJH pore size distribution in Figure 1b describes the small-mesopore region, whereas micropore volumes were quantified separately by the t-plot method (Section 3.3). Consistently, CH exhibited a BET surface area of 525 m^2^ g^−1^, a total pore volume of 0.216 cm^3^ g^−1^, and a micropore volume of 0.186 cm^3^ g^−1^ (Table 1). Despite their different biomass origins, CH and SB exhibited remarkably similar absolute micropore volumes (0.186 and 0.189 cm^3^ g^−1^, respectively), indicating that pyrolysis at 800 °C generated a comparable volume of microporous domains in both materials. However, their relative pore contributions differed substantially. Micropores accounted for 86.3% of the total pore volume in CH, compared with 63.4% in SB, primarily because SB developed a considerably larger total pore volume (0.298 vs. 0.216 cm^3^ g^−1^). The desorption-derived BJH curves show a stronger small-mesopore feature for SB than for CH. These distributions are method-dependent estimates and should be interpreted together with the total and micropore volumes, rather than as proof of identical characteristic pore sizes. The overall mean pore diameters were 16.5 Å for CH and 19.9 Å for SB, within or close to the micropore–mesopore boundary defined by IUPAC; these mean values should be distinguished from the maxima of the BJH mesopore-size distributions [42]. Because both materials were produced at 800 °C, the contrasting pore-volume contributions are consistent with precursor-dependent differences; the independent effect of carbonization temperature was not evaluated.

In contrast, SB exhibited higher N_2_ adsorption throughout the entire relative pressure range (Figure 1c), reaching approximately 194 cm^3^(STP) g^−1^ and displaying a more pronounced hysteresis loop. This behavior is consistent with a larger contribution of mesopores. The pore size distribution (Figure 1d) shows a substantially greater pore volume in the small-mesopore region than CH; implications for aqueous Hg(II) transport are considered alongside the kinetic results. These characteristics are reflected in its higher BET surface area (599 m^2^ g^−1^), larger total pore volume (0.298 cm^3^ g^−1^), and greater mean pore diameter (19.9 Å) relative to CH (16.5 Å) (Table 1). Although SB exhibited a lower micropore fraction (63.4%), its micropore volume remained comparable to that of CH, indicating that the additional porosity generated in SB is mainly associated with mesoporous development rather than an increase in micropore volume.

The differences between the biochars show that identical pyrolysis conditions did not produce the same porous structure, consistent with a contribution from precursor-dependent properties. Previous studies have shown that the physicochemical characteristics of biochars are largely controlled by the lignocellulosic composition and inorganic fraction of the precursor, which govern pore formation and structural rearrangement during carbonization [43,44].

### 2.2. Morphological Evolution and Pore Accessibility

Field-emission scanning electron microscopy coupled with energy-dispersive X-ray spectroscopy (FESEM–EDS) was used to compare the morphology and elemental distribution of the biochars produced at 800 °C. As shown in Figure 2 and Figure 3, CH and SB developed markedly different surface architectures, despite being obtained under identical thermal conditions [31,43].

CH exhibited a heterogeneous morphology with irregular laminar fragments, fractured particles, angular edges, and rough, compact domains (Figure 2a,b). The cross-sectional image revealed a relatively dense internal structure with dispersed voids and elongated discontinuities, which may reflect contraction or partial collapse of the biomass framework during devolatilization. This morphology agrees with previous reports on coffee husk-derived biochars, where pyrolysis produces amorphous carbon-rich structures with heterogeneous surfaces and limited long-range ordering [25]. The compact morphology observed for CH is also consistent with the N_2_ physisorption results, which showed a high micropore fraction (86.3%) and a smaller mean pore diameter. Therefore, although CH contains a large fraction of microporous adsorption domains, many of these sites may be located in confined regions that are less accessible to Hg(II) during adsorption.

In contrast, SB exhibited a more open and structurally preserved morphology (Figure 3a,b). Although the material consisted mainly of fragmented particles, several fragments retained well-defined, channel-like architectures inherited from the vascular tissue of the original sugarcane bagasse. The preservation of such channels after pyrolysis indicates that part of the lignocellulosic architecture remained stable during thermal conversion. Similar observations have been reported for sugarcane bagasse-derived biochars, where the fibrous and vascular nature of the precursor favors the formation of accessible porous networks and enhances adsorption performance in water treatment applications [29,30].

The EDS elemental maps further revealed differences in the distribution of inorganic constituents. In both biochars, carbon was homogeneously distributed throughout the analyzed particles (Figure 2c and Figure 3c), confirming its role as the principal structural component of the carbon matrix. Oxygen was also broadly distributed (Figure 2d and Figure 3d), confirming its detection in the analyzed regions; EDS alone cannot distinguish carbonaceous functional groups from oxygen-bearing mineral phases. In CH, calcium and silicon were detected across the entire particle surface with relatively uniform spatial distributions (Figure 2e,f), suggesting that both elements remained finely dispersed within the carbon matrix. Calcium and silicon were also distributed across the analyzed SB fragments (Figure 3e,f). These maps show the spatial co-occurrence of inorganic constituents and the carbonaceous matrix, while differences in elemental abundance are evaluated using XRF [43,45].

The XRD patterns (Figure 4) showed the coexistence of a poorly ordered carbonaceous matrix and crystalline inorganic phases in both biochars after pyrolysis at 800 °C. A broad diffraction feature in the 2θ = 20–30° region was observed together with sharper mineral reflections. In lignocellulosic biochars, such broad features are commonly associated with disordered or turbostratic carbon domains generated during thermal conversion, whereas the sharper reflections arise from crystalline mineral constituents concentrated in the solid residue during pyrolysis [43,46]. Therefore, the XRD patterns indicate that the carbonaceous fraction of CH and SB remained predominantly poorly ordered at 800 °C, while precursor-derived inorganic constituents persisted as discrete crystalline phases [45].

Despite being produced under identical pyrolysis conditions, CH and SB exhibited clearly different crystalline mineral assemblages. For SB, comparison with the PDF-4 Minerals database showed reflections consistent with quartz (SiO_2_; PDF 01-091-6090), including the intense reflection at 2θ = 26.5°, reflection at 49.95° and 67.88°. Additional reflections at 2θ = 29.25°, 31.54°, 34.19°, 36.03°, 39.31° and 59.79° were consistent with a calcium–potassium aluminosilicate phosphate phase assigned as Levantite [KCa_3_(Al_2_Si_3_)O_11_(PO_4_); PDF 00-071-0496]. The predominance of Si-containing phases was supported by the XRF results (Table 2), which identified Si as the major inorganic constituent of SB (41.20 wt%), followed by Ca (25.80 wt%) and Fe (8.28 wt%). The occurrence of these elements can be related to the mineral nutrition and growth conditions of the original sugarcane. Silicon is taken up from the soil solution mainly as monosilicic acid and subsequently deposited as silica within plant tissues, whereas K, Ca, and P are essential mineral nutrients absorbed during plant growth. In addition, part of the crystalline SiO_2_, as well as Al- and Fe-bearing mineral components, may originate from soil particles adhering to the sugarcane during cultivation, harvesting, and processing [47,48].

These elements are commonly associated with the inorganic or ash-forming fraction of lignocellulosic biomass and can become concentrated in biochar as volatile organic matter is removed during pyrolysis. Thus, the combined XRD and XRF results indicate that SB contains silica-rich crystalline domains together with Ca-, K-, Al-, and P-containing mineral phases derived from the inorganic constituents of the original sugarcane bagasse.

During pyrolysis, decomposition and volatilization of the lignocellulosic fraction increase the relative concentration of these non-volatile inorganic constituents in the remaining solid [49,50,51,52]. This enrichment has been reported specifically for sugarcane bagasse biochar, where thermal conversion increased the ash fraction and concentrated mineral constituents such as Ca, P, and Si [53,54]. Therefore, the simultaneous occurrence of Si, Ca, K, Al, P, and Fe in SB reflects the inorganic composition of the precursor.

In contrast, CH exhibited a distinct crystalline profile, with reflections consistent with Marialite and quartz. Peaks at 2θ = 22.91°, 29.29°, 39.33°, 43.13°, 47.42°, and 48.43° were assigned to a Marialite-type phase [Na_3_(Al_3_Si_3_)O_24_Cl; PDF 01-070-6155], whereas the reflection at 2θ = 26.36° showed overlapping contributions from quartz and Marialite. These crystalline phases coexist with the poorly ordered carbonaceous matrix, indicating the retention of mineral constituents after pyrolysis.

The XRF results (Table 2) revealed clear differences in the inorganic composition of the two biochars. Among the elements reported by XRF, CH was dominated by Ca (58.50 wt%), followed by Fe (11.10 wt%), K (8.51 wt%), Si (4.37 wt%), and Al (3.61 wt%), whereas SB was dominated by Si (41.20 wt%) and Ca (25.80 wt%), followed by Fe (8.28 wt%), K (7.69 wt%), and P (6.13 wt%). The predominance of Ca and K in CH is consistent with the mineral composition previously reported for coffee silverskin, in which these elements occur naturally as part of the inorganic fraction of the residue [55,56]. Coffee silverskin originates from the tegument surrounding the coffee bean and retains mineral nutrients accumulated by the plant during growth, including K and Ca, whose abundance depends on the mineral composition of the coffee tissues and cultivation conditions [57]. During pyrolysis, degradation of the organic fraction increases the relative contribution of these non-volatile inorganic constituents to the resulting biochar [58]. Therefore, the contrasting XRF profiles of CH and SB reflect both the different mineral composition of their respective precursors and the concentration of these constituents during thermal conversion.

### 2.3. FTIR Analysis

The FTIR spectra recorded before and after Hg(II) adsorption (Figure 5) showed changes in band shape and relative prominence in both biochars. The broad feature within 3600–3000 cm^−1^ is generally associated with O–H stretching from hydrogen-bonded hydroxyl groups, with possible contributions from retained moisture. This region exhibited changes in its profile, particularly for CH, although a consistent decrease was not evident for both materials. Because the spectra are vertically offset, the comparisons below describe changes relative to the local baselines rather than differences in absolute signal intensity [4,13].

For CH, the most evident modifications included a shallower feature near 1500 cm^−1^ and changes throughout the 1350–900 cm^−1^ region. SB exhibited more extensive attenuation of features across approximately 1750–1200 cm^−1^, together with modifications at lower wavenumbers. The band near 1750 cm^−1^ is compatible with C=O stretching, whereas the 1600–1500 cm^−1^ region may contain overlapping contributions from aromatic skeletal vibrations and oxygen-containing functionalities. These observations are consistent with changes in the chemical environments of biochar constituents following Hg(II) exposure, although they do not uniquely identify the functional groups responsible for adsorption [59].

The fingerprint region may contain overlapping contributions from organic functionalities and mineral phases. Bands within 1200–1000 cm^−1^ can arise from C–O vibrations and, where silica is present, Si–O–Si stretching. Consequently, these features cannot be assigned exclusively to either contribution [60]. The feature near 966 cm^−1^, tentatively attributed to Si–O stretching in surface silanol groups (Si–OH), became less pronounced in SB after adsorption, while changes in its profile were observed for CH. These variations are compatible with alterations in the local environment of silanol-containing sites, but do not establish their direct involvement in Hg(II) binding [61]. The band near 499 cm^−1^, potentially associated with silica-related bending vibrations, remained prominent after adsorption, particularly for SB, rather than showing a uniform attenuation [62].

### 2.4. Surface Charge Properties and Acid-Base Behavior (pH_p_ZC)

The pH-drift curves of CH and SB (Figure 6) exhibited similar profiles, with ΔpH increasing under acidic conditions, reaching a maximum at approximately pH 4, and subsequently decreasing toward zero as the initial pH increased. The ΔpH = 0 intercepts indicate pH_p_ZC values in the alkaline region (approximately 9–10) for both biochars. The initial and final pH values measured during adsorption (Table S1) remained below these pH_p_ZC estimates, suggesting positively charged surfaces under the experimental conditions.

Hg(II) speciation depends on pH and solution composition; hydrolyzed species may therefore participate in adsorption [63]. Therefore, the adsorption observed in the present study is more consistently interpreted as arising from specific interactions with surface sites rather thanfrom electrostatic attraction alone. In particular, complexation with oxygen-containing functionalities and interactions with mineral-associated sites are plausible pathways, consistent with previous studies on Hg(II) adsorption by biochars and with the changes observed here by FTIR and XPS after Hg(II) adsorption [15].

During the kinetic experiments, pH increased from 6.58 ± 0.09 to 7.62 ± 0.01 for CH and from 6.53 ± 0.11 to 7.19 ± 0.03 for SB (mean ± SD; *n* = 2; Table S1). The mean increases of 1.04 and 0.66 pH units, respectively, indicate an alkalinizing effect consistent with proton uptake by surface sites and/or release of alkaline constituents from the biochars [64]. Although CH produced the larger pH increase, SB exhibited greater Hg(II) uptake at 480 min, showing that the adsorption performance also depends on the textural and surface chemical differences discussed above.

### 2.5. Mechanistic Insights into Hg(II) Adsorption from XPS Analysis

High-resolution X-ray photoelectron spectroscopy (XPS) was employed to investigate the surface chemical composition of the carbon materials CH and SB before and after Hg adsorption. The analysis focused on the C 1s, O 1s, N 1s, and overlapping Hg 4f–Si 2p spectral regions to identify surface chemical modifications associated with mercury uptake and to assess the possible surface environments involved in the adsorption process.

The C 1s spectra of both materials are dominated by the main component centered at approximately 284.8 eV, assigned to C–C/C=C environments within the carbonaceous matrix. Contributions at higher binding energies can be associated with oxygen-containing carbon species, including C–O species around 286 eV, carbonyl groups (C=O) in the range of 287–288 eV, and carboxyl-type environments (O–C=O) near 288–289 eV. After Hg adsorption, no major changes are observed in the overall C 1s envelope, suggesting that Hg uptake does not substantially modify the surface carbon chemical environments.

More noticeable changes are observed in the O 1s region. The spectra contain contributions from several oxygen chemical environments associated with both the carbonaceous matrix and the inorganic fraction of the biochars. The lower-binding-energy region (~530.8–531.5 eV) may include contributions from carbonyl-type oxygen as well as oxygen associated with inorganic surface environments, whereas the higher-binding-energy region (~532.2–533.5 eV) may contain contributions from C–O and hydroxyl groups, Si–O-containing environments, and adsorbed water. Therefore, considering the mineralogical complexity of these materials, an unequivocal assignment of each O 1s component to a single functional group is not possible.

After Hg adsorption, a redistribution of the O 1s spectral envelope is observed for both CH and SB, particularly in the 532–533 eV region. These changes indicate modification of the local chemical environment of surface oxygen species and support the participation of oxygen-containing environments in Hg retention. However, the contribution of carbonaceous functional groups, mineral-associated oxygen, and adsorbed water cannot be completely resolved from the O 1s spectra alone.

The N 1s spectra exhibit a contribution centered around 399–400 eV, which can be attributed to nitrogen functionalities commonly found in carbon materials, such as pyridinic, pyrrolic or amine-type nitrogen species. No major changes in the position or shape of this signal were resolved after Hg adsorption. Thus, N 1s provides no clear evidence of substantial modification of nitrogen environments, although their participation in Hg retention cannot be excluded [62,65,66].

The most direct evidence of mercury uptake is observed in the Hg 4f spectral region after adsorption. The spectra show the characteristic Hg 4f spin–orbit doublet with the Hg 4f7/2 and Hg 4f5/2 peaks located at approximately 101–102 eV and 105–106 eV, respectively. The spin–orbit separation of about 4 eV is consistent with reported values for Hg 4f core levels. The binding energy of the Hg 4f7/2 peak near 101 eV is consistent with predominantly oxidized mercury, including Hg(II), on both surfaces [67]. No clear component attributable to metallic Hg^0^ near 100 eV was resolved under the investigated conditions; this observation does not exclude minor species below the resolution or sensitivity of the analysis [63,68].

In addition to the Hg 4f doublet, a contribution centered around ~103–104 eV is observed and assigned to the Si 2p core level, indicating the presence of a silica-containing mineral fraction within the carbon matrix. The Si 2p contribution is more pronounced in SB than in CH (Figure 7 and Figure 8), consistent with the Si-rich composition reported by XRF. However, the presence of this signal alone does not establish Hg binding to Si-containing sites [69,70].

Overall, the XPS results confirm the retention of mercury at the surface of both biochars and indicate that Hg remains predominantly in an oxidized state after adsorption. The changes observed in the O 1s region support the participation of oxygen-containing surface environments in Hg retention. For SB, the stronger Si 2p contribution, together with the XRD and XRF results, suggests that Si-containing mineral domains may provide additional oxygen-based interaction environments. Thus, the XPS results are consistent with Hg–O interactions involving both carbonaceous and mineral-associated oxygen sites, although the relative contribution of these environments cannot be determined from XPS alone.

### 2.6. Evaluation of Hg(II) Adsorption from Aqueous Solution

#### 2.6.1. Time-Dependent Adsorption Profiles and Uptake Dynamics

The kinetic profiles of Hg(II) adsorption on CH and SB (Figure 9) reveal not only differences in adsorption rate but also distinct time-dependent uptake patterns and Hg(II) uptake. SB exhibited a steep initial uptake during the first minutes of contact, followed by a progressive decrease in adsorption rate toward an operational plateau at 480 min. This rapid initial uptake is consistent with readily accessible adsorption sites at the solid–liquid interface or relatively short diffusion pathways. Similar behavior has been reported for hierarchically porous biochars, where improved pore accessibility and larger accessible pore volume enhance mass transfer and accelerate heavy-metal adsorption [71]. At 480 min, SB reached a Q_t_ of approximately 4.43 mg g^−1^, compared with approximately 2.88 mg g^−1^ for CH under identical experimental conditions, representing an approximately 53.8% higher Hg(II) uptake by SB at this contact time.

In contrast, CH showed a less abrupt initial increase and lower Q_t_ throughout the experiment. Its more gradual early uptake may reflect slower access to confined internal regions. Adsorption onto porous solids can involve both external mass transfer and intraparticle transport, whose relative contributions depend on the adsorbent and operating conditions [72]. An operational equilibrium time of 480 min was adopted for both biochars: the subsequent measurements at 600, 720, and 1440 min showed less than 5% variation in adsorption capacity relative to the value at 480 min (Figure 9). These later measurements confirmed the stability of Q_t_ beyond the interval used for kinetic fitting. Therefore, the slower uptake observed for CH is consistent with the textural results (Figure 1), which showed a higher micropore fraction and smaller mean pore diameter than SB.

The relationship between pore architecture and Hg(II) uptake becomes clearer when the kinetic results are considered alongside the BET and FESEM analyses. Although CH exhibited a predominantly microporous structure, the micropore volumes of the two biochars were comparable, indicating that differences in micropore abundance alone cannot explain their contrasting adsorption behavior. SB, however, exhibited a larger total pore volume, a greater contribution of wider pores, and preserved channel-like structures that may provide more accessible transport pathways toward internal adsorption domains. These characteristics could facilitate the movement of Hg(II) through the particles and promote the utilization of adsorption sites that would otherwise be less accessible within a predominantly microporous structure. In CH, the smaller mean pore diameter and comparatively compact morphology may impose greater mass-transfer limitations, contributing to the more gradual uptake described above. Thus, the higher Q_t_ achieved by SB at 480 min is consistent with the proposed role of pore accessibility. Pore connectivity and diffusion coefficients were not measured directly, so this explanation remains an interpretation of the combined textural, morphological, and kinetic evidence. This interpretation is consistent with studies showing that a more developed pore structure can enhance metal-ion transport within biochar particles [71,73]

#### 2.6.2. Fitting of Adsorption Kinetic Models

The kinetic parameters obtained by fitting the 0–480 min data with the PFO, PSO, Avrami, Elovich, and Weber–Morris models are summarized in Table S2, while the corresponding fits are presented in Figures S1 and S2. Among the evaluated models, Avrami and Elovich provided the best fits to the experimental data for both biochars, as evidenced by their higher coefficients of determination and lower reduced χ^2^ values. For SB, Avrami (R^2^ = 0.996; reduced χ^2^ = 0.0095) and Elovich (R^2^ = 0.989; reduced χ^2^ = 0.0216) outperformed PFO (R^2^ = 0.831) and PSO (R^2^ = 0.918). A similar trend was observed for CH, with Avrami (R^2^ = 0.985; reduced χ^2^ = 0.0152) and Elovich (R^2^ = 0.980; reduced χ^2^ = 0.018) providing better fits than PFO (R^2^ = 0.771) and PSO (R^2^ = 0.869). This ranking was also supported by the adjusted R^2^ values. The performance of Avrami and Elovich is consistent with kinetically complex adsorption behavior and surface heterogeneity [74]. However, the relatively large standard errors of the Avrami equilibrium capacity and rate constant for CH warrant caution when interpreting these parameters. Moreover, kinetic model fitting alone cannot establish the molecular adsorption mechanism, which requires complementary equilibrium, structural, and spectroscopic evidence [72,75,76]. The Weber–Morris model exhibited lower coefficients of determination (R^2^ = 0.503 for SB and 0.758 for CH) and nonzero intercepts (C = 2.48 ± 0.40 and 1.09 ± 0.21 mg g^−1^, respectively), suggesting that intraparticle diffusion was not the sole rate-controlling process. Overall, Avrami and Elovich provided the most suitable empirical descriptions of Hg(II) uptake under the investigated conditions, while the specific Hg(II)–surface interactions should be interpreted alongside the complementary physicochemical and spectroscopic evidence.

#### 2.6.3. Equilibrium Behavior, Thermodynamic Response and Affinity–Capacity Relationship

The equilibrium adsorption behavior of Hg(II) on CH and SB was evaluated at 293.15, 298.15, 308.15, 313.15, and 318.15 K using the Langmuir and Freundlich models. The corresponding parameters, including the Freundlich constants (Kf and 1/n) and the Langmuir maximum adsorption capacity (q_m_) and affinity constant (K_l_), are summarized in Table S3. Both models adequately described most of the experimental datasets, although their relative fitting performance varied with biochar type and temperature. For SB, Freundlich R^2^ values ranged from 0.957 to 0.995, whereas Langmuir R^2^ values ranged from 0.950 to 0.995. For CH, the corresponding ranges were 0.909–0.985 and 0.871–0.989, respectively. Notably, the Langmuir model provided particularly strong fits for SB between 298.15 and 318.15 K (R^2^ = 0.975–0.995) and for CH at 293.15 and 298.15 K (R^2^ = 0.975 and 0.989, respectively). Thus, neither model consistently outperformed the other across all experimental conditions. Rather than establishing a unique adsorption mechanism, the satisfactory performance of both models reflects the complexity of Hg(II) equilibrium behavior on chemically and structurally heterogeneous biochar surfaces [15,63].

The Langmuir parameters summarized in Table S3 revealed a clear temperature-dependent affinity–capacity relationship. The model-estimated maximum adsorption capacity (q_m_) of SB increased from 20.92 mg g^−1^ at 293.15 K to 45.05 mg g^−1^ at 318.15 K, whereas that of CH increased from 12.56 to 45.25 mg g^−1^ over the same temperature range. At 293.15 K, SB therefore exhibited a substantially greater model-estimated maximum capacity than CH, whereas this difference progressively decreased with increasing temperature, resulting in nearly identical q_m_ values at 318.15 K. In contrast, K_l_ decreased from 1.14 to 0.129 L mg^−1^ for SB and from 0.465 to 0.0854 L mg^−1^ for CH. Because q_m_ represents the theoretical maximum monolayer capacity predicted by the Langmuir model, whereas K_l_ reflects the apparent adsorbent–adsorbate affinity within this model, their opposite temperature trends should not be considered contradictory. These opposite temperature trends indicate that adsorption capacity and affinity represent distinct and complementary aspects of the equilibrium process. This distinction is particularly relevant when comparing adsorbents because a higher model-estimated saturation capacity does not necessarily imply stronger affinity for Hg(II), and overall adsorption performance may additionally depend on pore accessibility and mass-transfer pathways [77]. Nevertheless, at 318.15 K, the highest experimentally observed adsorption capacities were 31.95 mg g^−1^ for SB and 30.26 mg g^−1^ for CH, below the corresponding Langmuir-estimated q_m_ values. Since the experimental data do not conclusively establish a saturation plateau within the investigated concentration range, these q_m_ values should be interpreted as model-dependent estimates rather than experimentally verified saturation capacities [78].

The Freundlich parameters summarized in Table S3 provide complementary information on the equilibrium behavior of the two biochars. Kf ranged from 4.57 to 8.94 for SB and from 3.55 to 4.20 for CH, whereas 1/n ranged from 0.333 to 0.652 and from 0.334 to 0.739, respectively. In all cases, 1/n remained between 0 and 1, indicating favorable Hg(II) adsorption within the investigated concentration range. Variations in Kf and 1/n with temperature further indicate temperature-dependent changes in apparent adsorption capacity and adsorption intensity. The ability of the Freundlich model to describe the experimental data is consistent with the physicochemical heterogeneity identified by FTIR, XPS, XRD, XRF, and FESEM–EDS, which revealed the coexistence of carbonaceous domains, oxygen-containing functionalities, and inorganic mineral phases capable of providing adsorption environments with different characteristics [15,63]. However, the strong performance of the Langmuir model at several temperatures indicates that such heterogeneity does not preclude Langmuir-type equilibrium behavior within the investigated concentration range.

Thermodynamic analysis, with the corresponding parameters summarized in Tables S4 and S5, indicated an apparent exothermic response for both biochars under the adopted Langmuir-based equilibrium convention. CH exhibited ΔH° = −52.8 kJ mol^−1^ and ΔS° = −50.9 J mol^−1^ K^−1^, with a Van’t Hoff R^2^ of 0.963 (Figure S3), whereas SB exhibited ΔH° = −71.4 kJ mol^−1^, ΔS° = −107.7 J mol^−1^ K^−1^, and R^2^ = 0.975 (Figure S4). The negative ΔH° values indicate an exothermic Langmuir-based equilibrium response for both biochars, consistent with the progressive decrease in K_l_ with increasing temperature. Exothermic Hg(II) adsorption has also been reported for carbonaceous adsorbents, although both exothermic and endothermic responses have been observed depending on precursor composition, surface chemistry, and material modification [79]. The negative ΔS° values indicate a decrease in the apparent degree of disorder at the solid–solution interface upon Hg(II) adsorption, while the negative ΔG° values throughout the investigated temperature range indicate thermodynamically favorable adsorption. Nevertheless, these thermodynamic quantities describe the overall equilibrium response and should not be interpreted independently as evidence of a specific molecular adsorption mechanism.

Finally, the affinity–capacity relationship provides an important link between the equilibrium results and the structural characteristics of the two biochars. SB combined higher q_m_ and K_l_ values at the lower temperatures with the faster Hg(II) uptake observed in the kinetic experiments, whereas CH exhibited lower model-estimated capacities at the lower temperatures and a more gradual kinetic profile. This behavior is consistent with the greater total pore volume, greater contribution of wider pores, and preserved channel-like morphology of SB, which may facilitate access to internal adsorption domains and reduce mass-transfer limitations [77,80]. However, as temperature increased, the Langmuir capacities of the two biochars converged despite the progressive decrease in K_l_, demonstrating that maximum adsorption capacity alone does not fully describe adsorbent performance.

### 2.7. Factors Affecting Hg(II) Adsorption

The results of this study demonstrate that Hg(II) adsorption by CH and SB cannot be explained by a single material property, but rather by the combined influence of pore architecture, surface chemistry, mineral composition, and mass-transfer behavior. Although both biochars were produced under identical pyrolysis conditions at 800 °C, their precursor-dependent characteristics resulted in distinct adsorption behavior. This observation is consistent with previous studies showing that biochar physicochemical properties are strongly influenced by feedstock composition, mineral content, and the transformations occurring during pyrolysis [13,43,81].

Textural and morphological analyses revealed marked differences in the pore architecture of the two biochars. CH exhibited a predominantly microporous structure, with a micropore fraction of 86.3% and a smaller mean pore diameter, whereas SB showed a higher BET surface area, larger total pore volume, and a greater contribution of wider pores. Notably, the micropore volumes of CH and SB were comparable, indicating that the higher Hg(II) uptake of SB at 480 min cannot be attributed simply to a greater abundance of micropores [4]. FESEM observations provided complementary morphological evidence: SB preserved numerous channel-like cavities consistent with the vascular architecture of sugarcane bagasse, whereas CH exhibited a comparatively compact and less organized surface with fewer and more irregular visible cavities (Figure S5). This distinction may be particularly relevant for Hg(II) adsorption because aqueous Hg species must be transported through the pore network before reaching internal adsorption environments. Previous studies have demonstrated that Hg(II) can progressively penetrate biochar particles and that its aqueous speciation can influence access to intraparticle sorption sites [82,83]. Thus, the higher Hg(II) uptake of SB at 480 min may be associated with greater access to internal adsorption environments through wider pores and preserved channels, consistent with evidence that Hg can penetrate biochar particles [83,84].

Mineralogical and elemental analyses further demonstrated important precursor-dependent differences. XRD identified quartz and Levantite as the main crystalline phases in SB, consistent with its high Si content determined by XRF, whereas CH was characterized by Marialite and quartz and exhibited substantially higher Ca content. These differences contribute to the chemical heterogeneity of the biochars by generating distinct carbonaceous and mineral-associated adsorption environments [15]. Spectroscopic evidence complements this interpretation. FTIR and XPS showed changes in oxygen-containing environments after Hg(II) adsorption in both materials, consistent with previous studies associating hydroxyl, carboxyl, carbonyl, and related oxygen-containing functionalities with Hg(II) retention by biochars [66]. As described in Section 2.3, CH showed changes near 1500 cm^−1^ and across 1350–900 cm^−1^, whereas SB showed more extensive attenuation across approximately 1750–1200 cm^−1^ and additional changes at lower wavenumbers. Overlapping organic and mineral vibrations prevent unique assignment of these changes to individual adsorption sites. The stronger Si 2p signal of SB, together with its Si-rich mineral composition and the redistribution of O 1s components after Hg adsorption, suggests that mineral-associated oxygen environments may complement the oxygen-containing sites of the carbon matrix. Similar Hg(II) interactions with silanol and aluminol environments have been reported for silica- and aluminosilicate-based adsorbents [15,63,85]. The Hg 4f7/2 signal at approximately 101–102 eV is consistent with predominantly oxidized mercury after adsorption, but does not identify a specific Hg–O bonding configuration. These complementary observations form the experimental basis for the adsorption mechanism proposed in Scheme 1. Despite their similar pH_p_zc values, CH and SB exhibited markedly different Hg(II) adsorption performances, indicating that differences in net surface charge alone cannot explain their contrasting behavior. Instead, this observation reinforces the importance of the accessibility and chemical nature of the available adsorption environments in controlling Hg(II) uptake [69].

Kinetic analysis provides additional support for the contribution of multiple processes to Hg(II) uptake. The Avrami model provided the highest statistical agreement for both biochars, followed closely by Elovich, whereas PFO and PSO showed poorer fits [86]. The substantially lower agreement obtained with the Weber–Morris model, particularly for SB, indicates that intraparticle diffusion alone cannot account for the overall adsorption kinetics [72,86]. These statistical differences should not be interpreted as direct evidence of a specific adsorption mechanism; rather, they are consistent with Hg(II) uptake involving more than a single rate-controlling process [63]. Wu et al. reported that Cd(II) uptake by hierarchically porous biochars involved both external and internal mass transfer, whereas Pb(II) adsorption was controlled by intraparticle diffusion, illustrating that transport limitations can differ between metal–biochar systems [73].

Equilibrium analysis further highlights the distinction between adsorption capacity and affinity. Both Langmuir and Freundlich provided satisfactory descriptions of most experimental datasets, although their relative performance varied with biochar type and temperature. The Langmuir-estimated q_m_ increased with temperature for both biochars, whereas K_l_ progressively decreased, showing that a higher theoretical saturation capacity does not necessarily correspond to greater apparent Hg(II) affinity. This distinction is consistent with Thomas et al. (2020), who identified maximum sorption capacity and affinity as complementary but distinct attributes of metal adsorption by biochars. Accordingly, the equilibrium response of CH and SB should be interpreted by considering capacity and affinity jointly rather than either parameter in isolation [87].

Thermodynamic analysis provides an additional energetic perspective. The Langmuir-based apparent equilibrium response was exothermic for both biochars, with ΔH° values of −52.8 kJ mol^−1^ for CH and −71.4 kJ mol^−1^ for SB, consistent with the overall decrease in K_l_ with increasing temperature. Negative ΔG° values throughout the investigated temperature range indicate thermodynamically favorable adsorption, whereas negative ΔS° values indicate increased ordering at the solid–solution interface upon Hg(II) retention. Notably, different thermodynamic responses have been reported even for Hg(II) adsorption by carbonaceous materials derived from similar agricultural residues. Homagai et al. 2022 reported endothermic Hg(II) adsorption on charred sugarcane bagasse (ΔH° = +52.1 kJ mol^−1^), contrasting with the exothermic behavior observed here for SB [36]. This difference highlights the sensitivity of adsorption energetics to the physicochemical characteristics developed during carbonization and to the experimental conditions.

Taken together, the results are consistent with contributions from pore accessibility, mass transfer, adsorption affinity, and chemically diverse adsorption environments to the contrasting Hg(II) uptake of CH and SB. The predominantly microporous architecture of CH may impose greater accessibility constraints, whereas the larger total pore volume, greater contribution of wider pores, and preserved channel-like morphology of SB provide more favorable transport pathways toward internal regions of the particles. In SB, these structural characteristics coexist with a chemically diverse surface and a Si-rich mineral fraction that may provide additional oxygen-containing environments for Hg(II) interaction. This interpretation agrees with previous studies showing that metal adsorption by biochar depends on the combined influence of pore architecture, accessible surface area, functional groups, and mineral components [14,84]. Therefore, the higher Hg(II) uptake of SB at 480 min is most consistently associated with a more favorable combination of pore accessibility, mass-transfer pathways, and chemically diverse adsorption environments rather than with any single physicochemical property.

#### Mechanistic Interpretation of Hg(II) Adsorption on CH and SB Biochars

The proposed Hg(II) adsorption mechanism (Scheme 1) is supported by the complementary textural, spectroscopic, and mineralogical evidence obtained in this study. Together with the kinetic results, BET and FESEM support the hypothesis that pore architecture influences access to internal adsorption domains: the micropore-dominated structure of CH may impose greater diffusional constraints, whereas the wider pores and preserved channel-like morphology of SB may facilitate transport toward reactive sites. Once these sites are reached, FTIR and XPS indicate an important contribution from oxygen-containing surface environments. In CH, FTIR changes near 1500 cm^−1^ and across 1350–900 cm^−1^, together with redistribution of the O 1s envelope at 532–533 eV, are consistent with changes in oxygen-containing environments. However, the overlapping FTIR bands and O 1s contributions do not resolve individual functional groups or distinguish their contributions from mineral-associated oxygen.

For SB, carbonaceous and mineral-associated oxygen environments are plausible contributors to Hg(II) retention. As described in Section 2.3, FTIR changes extended across approximately 1750–1200 cm^−1^ and into lower-wavenumber regions, where organic and mineral vibrations overlap. This behavior is consistent with XRD, which identified quartz and Levantite [KCa_3_(Al_2_Si_3_)O_11_(PO_4_)] as the main crystalline phases in SB, and with XPS, where the Si 2p contribution at approximately 103–104 eV was substantially stronger than in CH. Together with the changes observed in the O 1s region after Hg adsorption, these results suggest that oxygen environments associated with the silica- and aluminosilicate-rich mineral fraction may complement the oxygen-containing sites of the carbon matrix. This interpretation is supported by previous studies showing that silanol (≡Si–OH) and aluminol (≡Al–OH) sites on aluminosilicate surfaces can participate in Hg(II) retention through surface complexation [88], while Hg(II) adsorption on silica has been associated with interactions between hydrolyzed Hg species and surface silanol groups [89]. Recent spectroscopic evidence for silica-based adsorbents further shows that changes in O 1s and Si 2p environments during Hg(II) uptake can be associated with the participation of Si–OH and Si–O–Si environments [70]. In contrast, CH contained Marialite and quartz as its principal crystalline phases but showed a much weaker Si contribution. The Hg 4f spectra are consistent with predominantly oxidized mercury after adsorption, without a clearly resolved metallic-Hg component. Specific Hg–O bonding configurations and the relative contributions of organic and mineral sites remain unconfirmed.

## 3. Material and Methods

### 3.1. Reagents

All reagents used in this study were of analytical grade and used without further purification. Nitric acid (HNO_3_, 65%), analytical grade (ISO), was supplied by PanReac AppliChem (ITW Reagents). Mercury (II) chloride (HgCl_2_), analytical grade (ACS), also obtained from PanReac AppliChem (ITW Reagents), was used as the precursor for Hg (II) solution preparation. All solutions were prepared using deionized water.

### 3.2. Synthesis of Biochar

Two agro-industrial residues from the Amazon region were used as biochar precursors: the perisperm (silver skin) fraction of coffee husk (CH) and sugarcane bagasse (SB). The raw biomasses were first washed with deionized water to remove adhering impurities, then oven-dried at 60 °C for 48 h until a constant weight. The dried materials were mechanically ground and sieved to obtain particles smaller than 500 µm.

The samples were placed in open ceramic crucibles and subjected to direct pyrolysis in a programmable muffle furnace under oxygen-limited conditions. Carbonization was carried out at 800 °C with a heating rate of 4 °C min^−1^ and a residence time of 2 h. After pyrolysis, the furnace was allowed to cool naturally to below 50 °C with the chamber closed.

The resulting biochars were washed with deionized water at a solid-to-liquid ratio of 1:10 (*w*/*v*). The suspensions were stirred for 24 h, allowed to settle for 6 h, and the supernatant was removed. This procedure was repeated three times.

The washed biochars were dried at 60 °C until constant weight, ground using a mortar, and sieved through a 250 µm mesh. The resulting materials were labeled as CH and SB and used for subsequent adsorption experiments and physicochemical characterization.

### 3.3. Characterization Methods

The morphology and elemental distribution of the biochars were analyzed by field-emission scanning electron microscopy coupled with energy-dispersive X-ray spectroscopy (FESEM–EDS) using a dual-beam FIB–FESEM system (Scios 2 LoVac, Thermo Fisher Scientific, USA) equipped with an UltraDry EDS detector (129 eV resolution, 30 mm^2^ active area). Images were acquired using secondary electron and backscattered electron detectors to evaluate surface morphology, pore structure, and mineral distribution, while elemental mapping was performed to determine the spatial distribution of major elements on the biochar surface.

The bulk elemental composition was determined by X-ray fluorescence spectroscopy (XRF) using a NEX QC+ QuantEZ spectrometer. Measurements were conducted using tube voltages of 6.5, 30, and 50 kV for light, medium, and heavy elements, respectively, with an acquisition time of 30 s for each analytical condition. These measurements were used to quantify the major inorganic constituents present in the materials.

The crystalline structure of the biochars was investigated by X-ray diffraction (XRD) using a Miniflex diffractometer (Rigaku, Japan) operated at 40 kV and 30 mA and equipped with a D/teX Ultra detector. Diffraction patterns were collected using Cu Kα radiation (λ = 1.5405 Å) with a scan rate of 10° min^−1^ and a step size of 0.01° over a 2θ range from 2° to 90°. The obtained diffractograms were used to identify crystalline mineral phases and evaluate the degree of structural ordering of the carbon matrix.

The surface functional groups of the biochar samples were characterized by Fourier-transform infrared (FTIR) spectroscopy using a Shimadzu IRSpirit-1S FTIR spectrophotometer (Shimadzu Corporation, Kyoto, Japan). The samples were analyzed using the KBr pellet method. Briefly, the finely ground samples were mixed with spectroscopic-grade potassium bromide (KBr), homogenized, and compressed into pellets prior to analysis. The point of zero charge (pH_PZC_) was established following the solid addition method [90].

Surface chemical composition was analyzed by X-ray photoelectron spectroscopy (XPS) using an ACenteno XPS/ISS/UPS platform (SPECS, Germany) equipped with a monochromatic Al Kα X-ray source (FOCUS 500) operated at 200 W and a hemispherical energy analyzer with a 2-CMOS detector. Survey spectra were collected at a pass energy of 100 eV, whereas high-resolution spectra were recorded at 20 eV. Surface charge compensation was achieved using a Flood Gun system. High-resolution spectra were acquired in the C 1s, O 1s, N 1s, Hg 4f, and Si 2p regions to evaluate changes in surface chemistry associated with Hg(II) adsorption and to identify the chemical environments involved in the adsorption process.

The textural properties of the biochars were determined from N_2_ adsorption–desorption isotherms measured at approximately −198 °C for both biochars using a 3Flex analyzer (Micromeritics, USA). Prior to analysis, samples were degassed under vacuum at 90 °C for 1 h followed by 150 °C for 12 h. The specific surface area (S_(_BET_)_) was estimated using the Brunauer–Emmett–Teller (BET) method, with the fitting regions evaluated following the Rouquerol criteria [91]. Micropore volume (Vμp), micropore surface area (Sμp), and external surface area (Sext) were obtained using the t-plot method with the Harkins–Jura thickness equation. The reported thickness intervals were 4.48–5.51 Å for SB and 4.47–5.55 Å for CH. The pore-size distributions presented in Figure 1 were obtained using the Barrett–Joyner–Halenda (BJH) method applied to the desorption branch, with the Harkins–Jura thickness equation and Faas correction. Total pore volumes were taken from the adsorption branch at P/P_0_ ≈ 0.99 for both biochars, and mean pore diameters were calculated as 4V/A using these total pore volumes and the BET surface areas.

### 3.4. Batch Adsorption Experiments and Kinetic Modelling for Synthesized Chars

Hg(II) adsorption was evaluated through comparative batch experiments to determine adsorption kinetics and equilibrium behavior of the biochars. A 1000 mg L^−1^ Hg(II) stock solution was prepared by dissolving analytical-grade HgCl_2_ in deionized water and subsequently diluted to obtain working solutions.

Total mercury (Hg) was quantified following the EPA Method 7473 [92], using thermal decomposition coupled with atomic absorption spectrometry at 253.7 nm with Zeeman background correction. Analyses were performed using a Lumex RA-915 LAB (Lumex Instruments, St. Petersburg, Russia), and signal acquisition and integration were carried out using RAPID software.

For each analysis, 150 µL of sample was introduced into pre-cleaned quartz boats containing mercury-free activated carbon (Lumex Instruments, St. Petersburg, Russia) previously calcined at 850 °C to eliminate potential contamination. The quartz boats were transferred into the atomizer furnace, where the samples were thermally decomposed at 850 °C. The released mercury, reduced to its elemental form (Hg^0^), was subsequently detected in the analytical cell, which was also preheated to 850 °C. Quantification was performed using external calibration curves prepared from a mercury standard solution (Hg(NO_3_)_2_, 1000 mg L^−1^, Certipur^®^, Supelco). Three linear calibration ranges were established (0.2–20 ng, 12.5–750 ng, and 500–10,000 ng Hg), all excellent linearity (R^2^ ≥ 0.9998). The detection limit (LOD) was 1.41 µg L^−1^ and was calculated from the standard deviation of the analytical signal obtained from nine blank samples. Quality-control solutions containing 5, 10, and 50 µg L^−1^ Hg were prepared from (Hg(NO_3_)_2_ standard, yielding a mean recovery of 98.53%, with individual recoveries ranging from 78.9% to 115% (n = 10). In addition, blanks consisting of a quartz boat containing mercury-free activated carbon were analyzed after every 10 samples, and Hg concentrations in all blanks remained below the LOD.

For kinetic experiments, an adsorbent dose of 200 mg L^−1^ and an initial Hg (II) concentration of 1 mg L^−1^ (expressed as elemental mercury mass) were used in closed borosilicate-glass Erlenmeyer flasks. The systems were maintained under agitation at 160 rpm and 25 ± 1 °C, without external pH adjustment or control. The initial pH ranged from 6.45 to 6.64 in the paired kinetic measurements reported in Table S1. The initial and final pH values were measured to assess changes during the adsorption experiments. Aliquots of approximately 500 µL were withdrawn over a time range of 0–1440 min, filtered (0.22 µm), and acidified with HNO_3_ prior to mercury quantification. The cumulative volume removed during successive sampling was considered when calculating the adsorption capacity at each sampling time. An operational equilibrium time of 480 min was selected for both biochars, based on less than 5% variation in Q_t_ at the three subsequent measurements (600, 720, and 1440 min) relative to Q_t_ at 480 min.

Kinetic data from 0 to 480 min were fitted using non-linear least-squares regression with pseudo-first-order (PFO) (Text S1) pseudo-second-order (PSO) (Text S2), Avrami (Text S3), Elovich (Text S4), and Weber–Morris models (Text S5) [12,93,94,95].

For adsorption isotherms, Hg (II) solutions with initial concentrations of 1, 5, 10, 30, and 50 mg L^−1^ were used, maintaining a constant adsorbent dose (200 mg L^−1^) and the same experimental conditions described above. Suspensions were maintained in contact for 480 min before sampling, based on the operational plateau identified in the kinetic experiments. Samples were then filtered and analyzed following the same procedure used for kinetic experiments. The resulting data were used to evaluate adsorption isotherm models (Texts S6 and S7).

Thermodynamic parameters were estimated from the temperature dependence of the Langmuir equilibrium constant. The Langmuir constant (K_l_) was converted into a dimensionless apparent equilibrium constant (Kc) using a dilute-solution mole-fraction convention with water as the solvent and a fixed conversion factor of 55.5 mol L^−1^, which was subsequently used to calculate the Gibbs free-energy change (ΔG°) and to determine the enthalpy (ΔH°) and entropy (ΔS°) changes using the Van’t Hoff relationship. The complete equations, conversion factors, units, and supporting references are provided in Text S8 of the Supplementary Materials [70,96,97].

## 4. Conclusions

This study demonstrates that precursor biomass strongly influences the physicochemical properties and Hg(II) adsorption performance of biochars produced under identical pyrolysis conditions (800 °C). The direct comparison of non-activated coffee husk (CH) and sugarcane bagasse (SB) biochars shows that differences in Hg(II) uptake cannot be explained by a single material property but are consistent with the combined influence of pore architecture, surface chemistry, mineral composition, and mass-transfer behavior. The larger pore volume, greater contribution of wider pores, and preserved channel-like morphology of SB may facilitate access to internal adsorption environments compared with the predominantly microporous structure of CH. FTIR and XPS support the involvement of oxygen-containing environments in Hg(II) retention, while the Si-rich mineral fraction of SB may provide additional interaction environments, although a specific Hg–mineral bonding mechanism was not established.

The kinetic and equilibrium results further indicate that adsorption behavior reflects multiple contributing processes, and that model-estimated capacity and apparent affinity should be considered separately. Thermodynamic analysis indicated favorable and exothermic adsorption for both biochars under the adopted apparent-equilibrium convention. At 480 min under the investigated kinetic conditions, SB achieved approximately 53.8% higher Hg(II) uptake than CH. These findings support the potential of sugarcane bagasse biochar as a biomass-derived adsorbent for mercury removal from aqueous systems, warranting further evaluation under environmentally relevant water conditions.

## Figures and Tables

**Figure 1:**
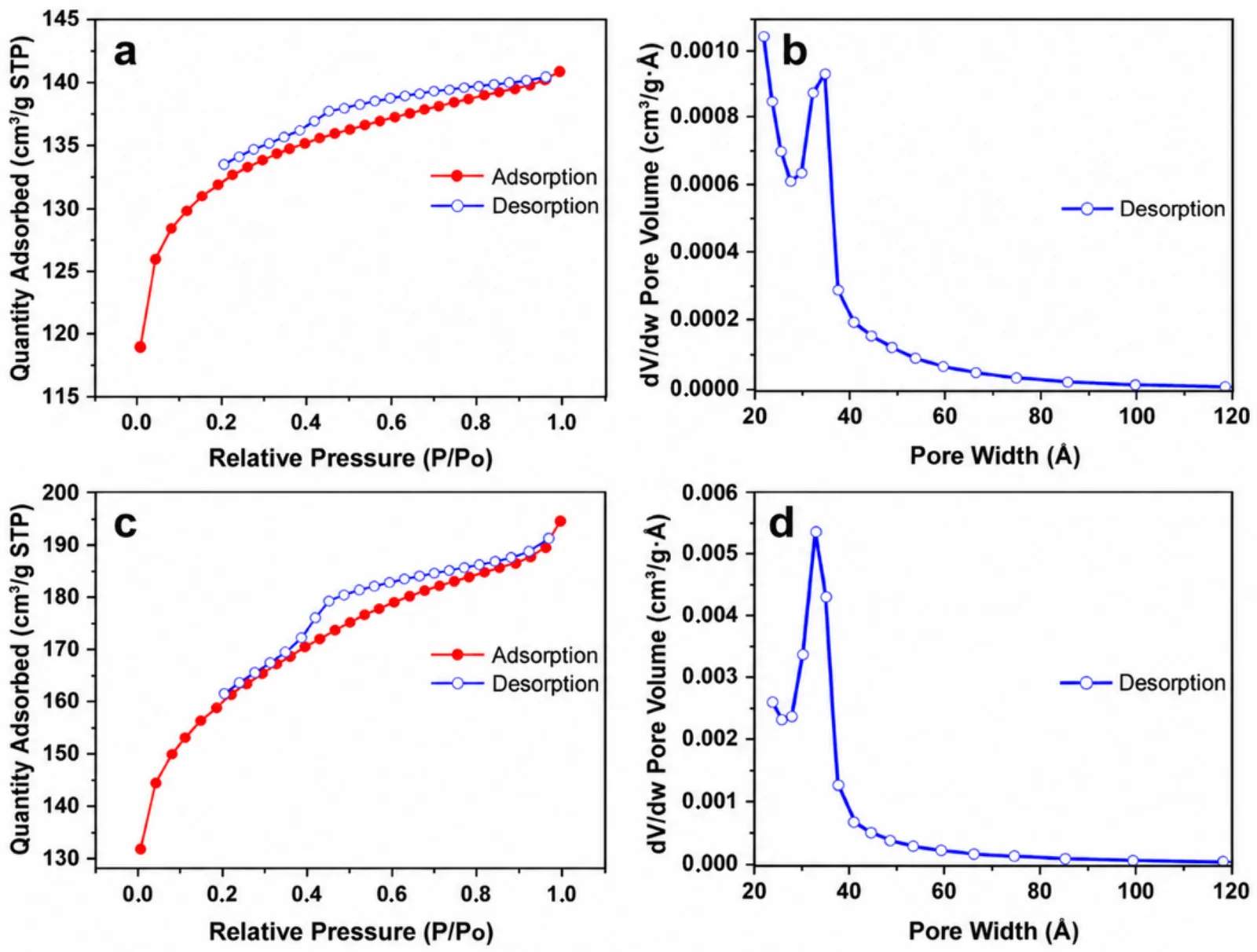
N_2_ adsorption–desorption isotherms and pore size distributions of coffee husk (CH) and sugarcane bagasse (SB) biochars obtained at approximately −198 °C. (**a**) Adsorption–desorption isotherm of CH; (**b**) pore size distribution of CH; (**c**) adsorption–desorption isotherm of SB; and (**d**) pore size distribution of SB. The pore size distributions shown in panels (**b**,**d**) were calculated using the BJH method applied to the desorption branch.

**Figure 2:**
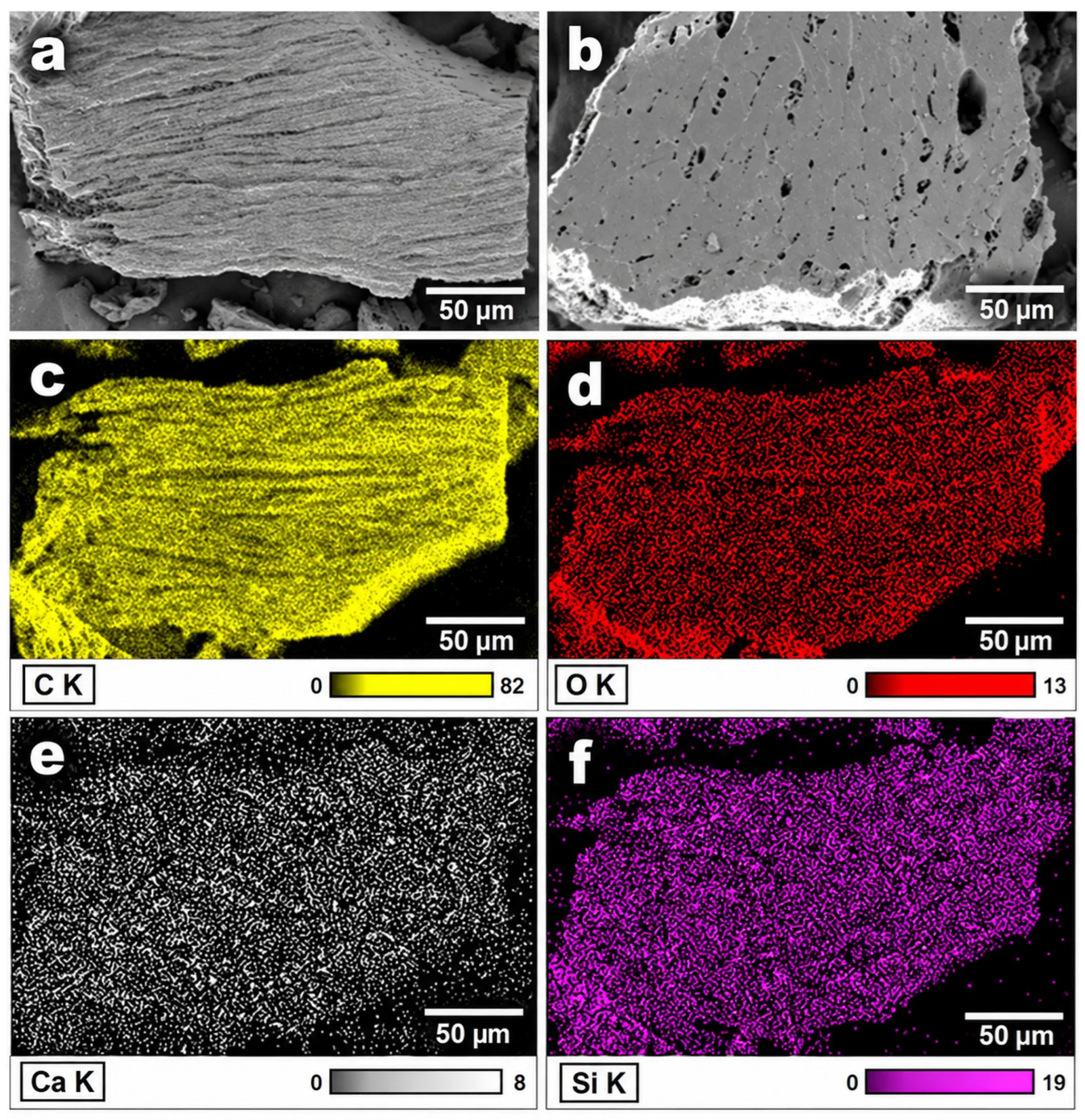
FESEM micrographs and EDS elemental mapping of coffee husk biochar (CH) produced at 800 °C. (**a**,**b**) Surface morphology; (**c**–**f**) elemental distribution maps of C, O, Ca, and Si, respectively.

**Figure 3:**
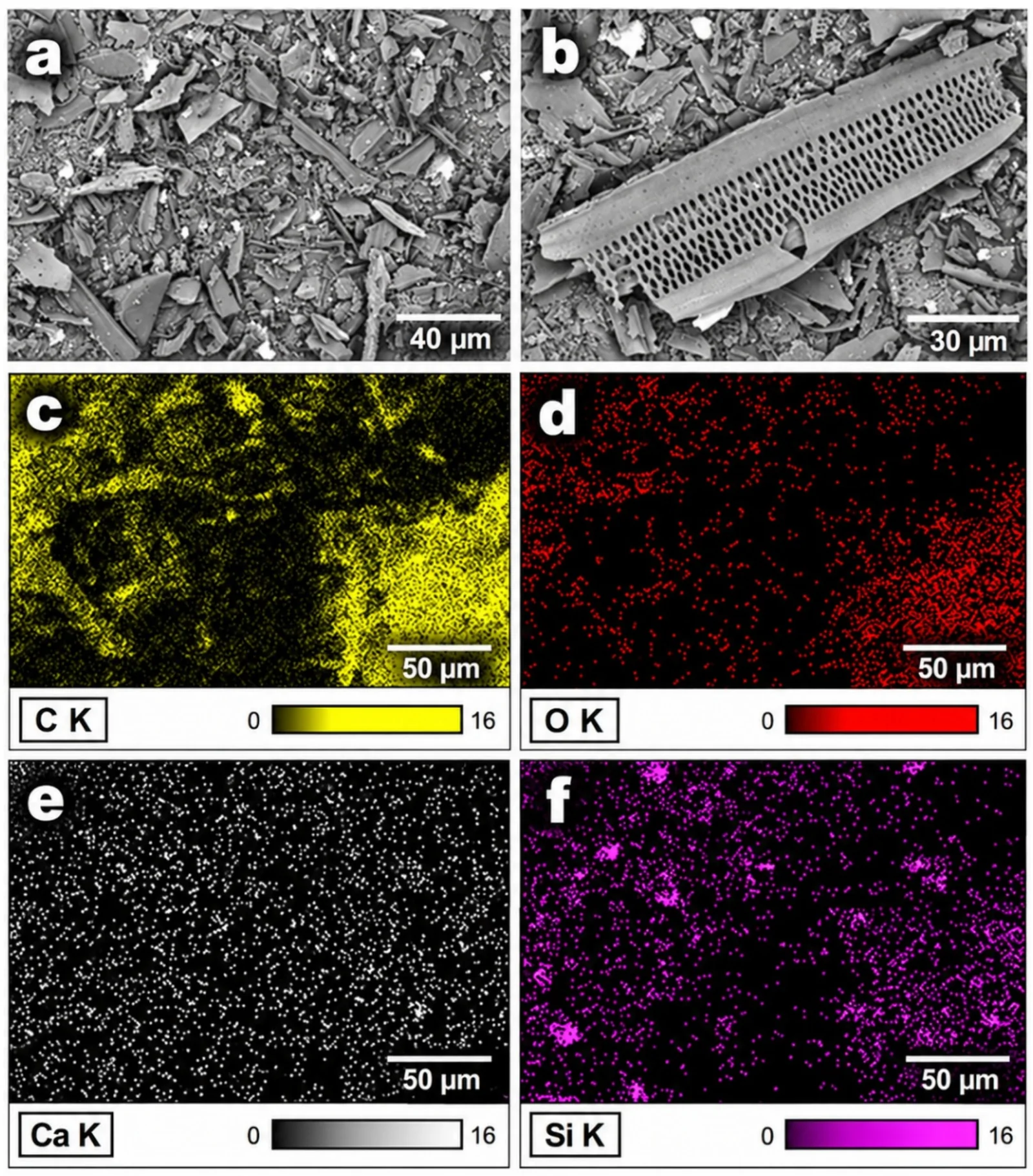
FESEM micrographs and EDS elemental mapping of sugarcane bagasse biochar (SB) produced at 800 °C. (**a**,**b**) Surface morphology; (**c**–**f**) elemental distribution maps of C, O, Ca, and Si, respectively.

**Figure 4:**
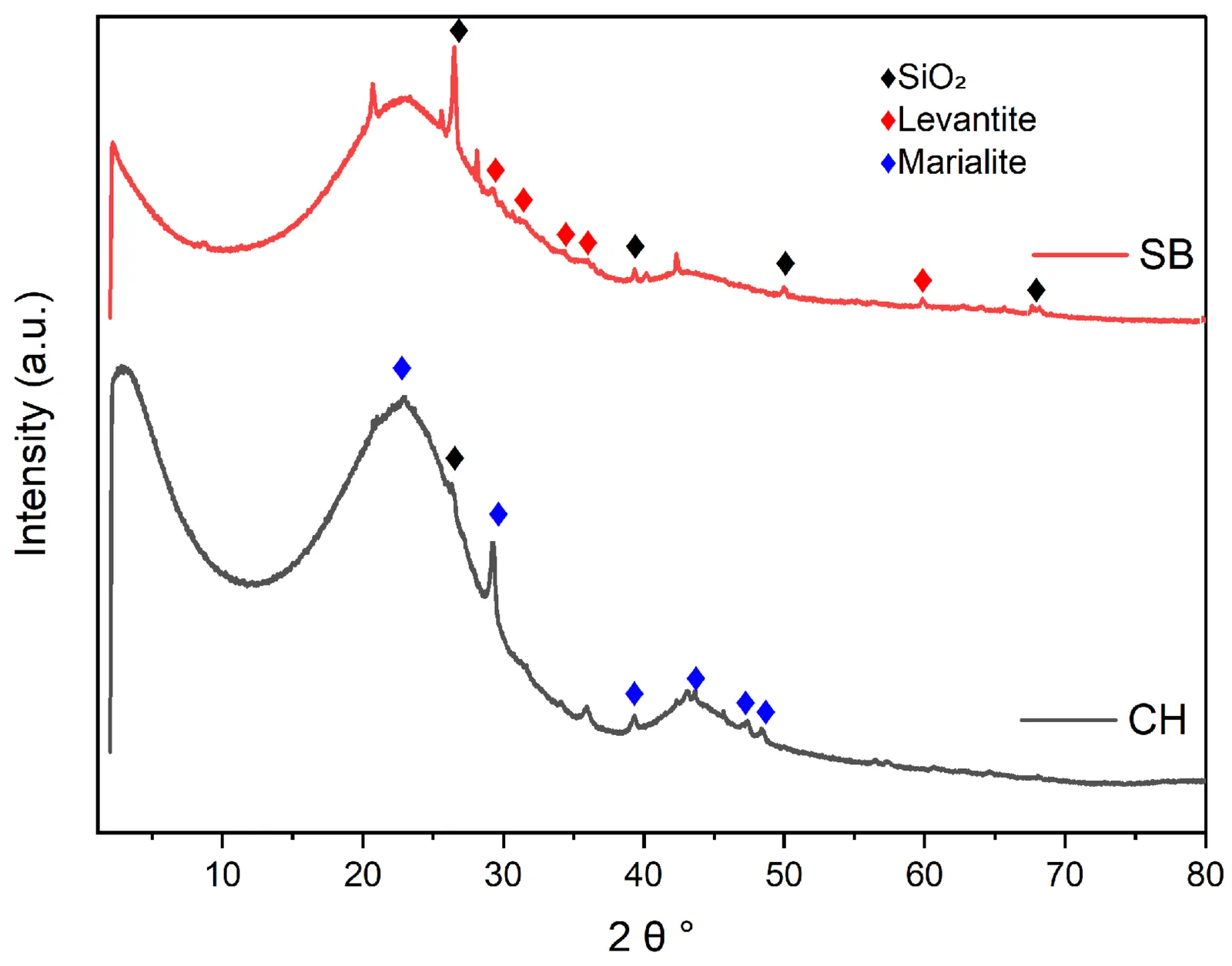
XRD patterns of SB and CH biochars. Symbols indicate quartz (SiO_2_, ♦), Levantite ♦, and Marialite ♦.

**Figure 5:**
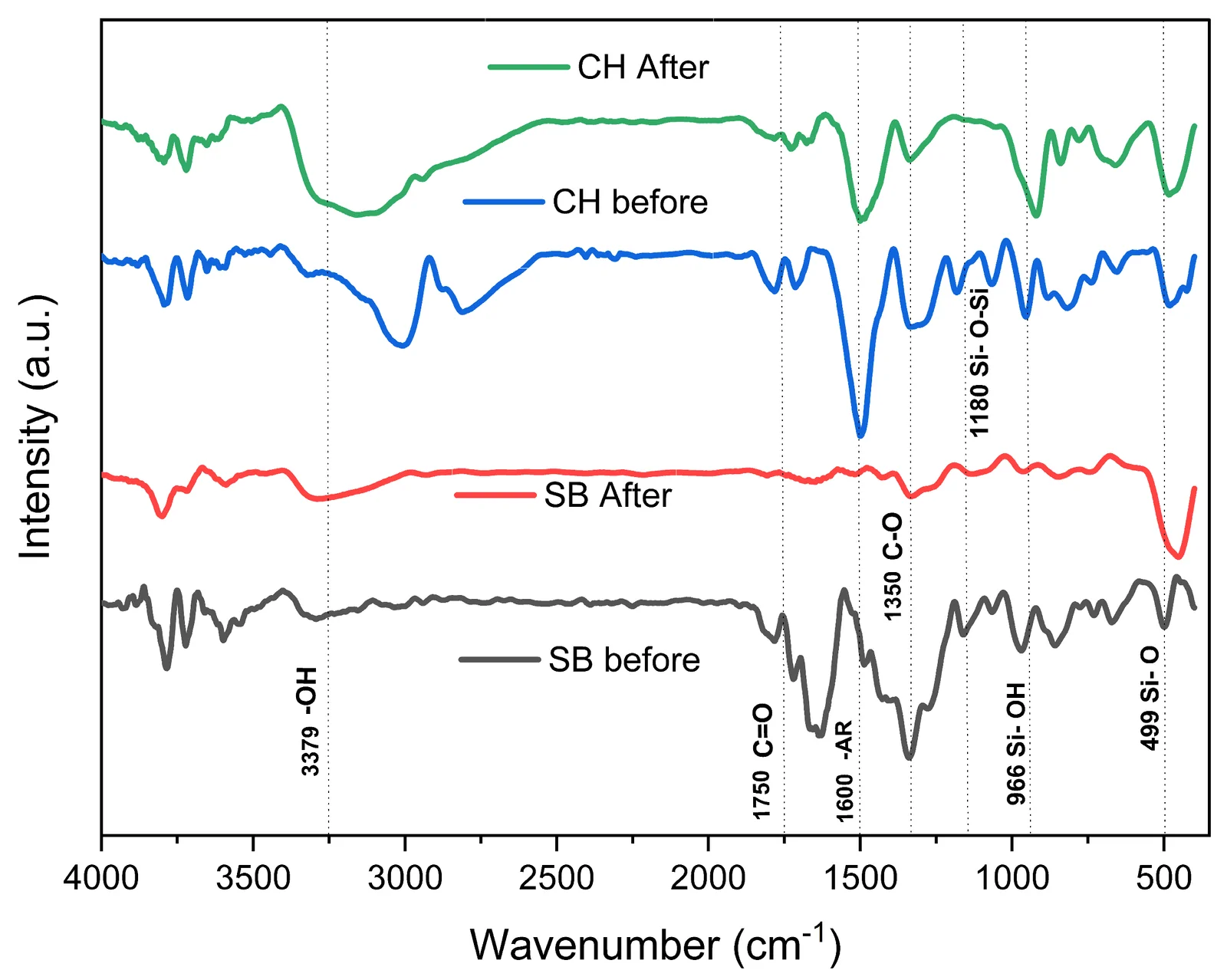
FTIR spectra of CH and SB before and after Hg(II) adsorption.

**Figure 6:**
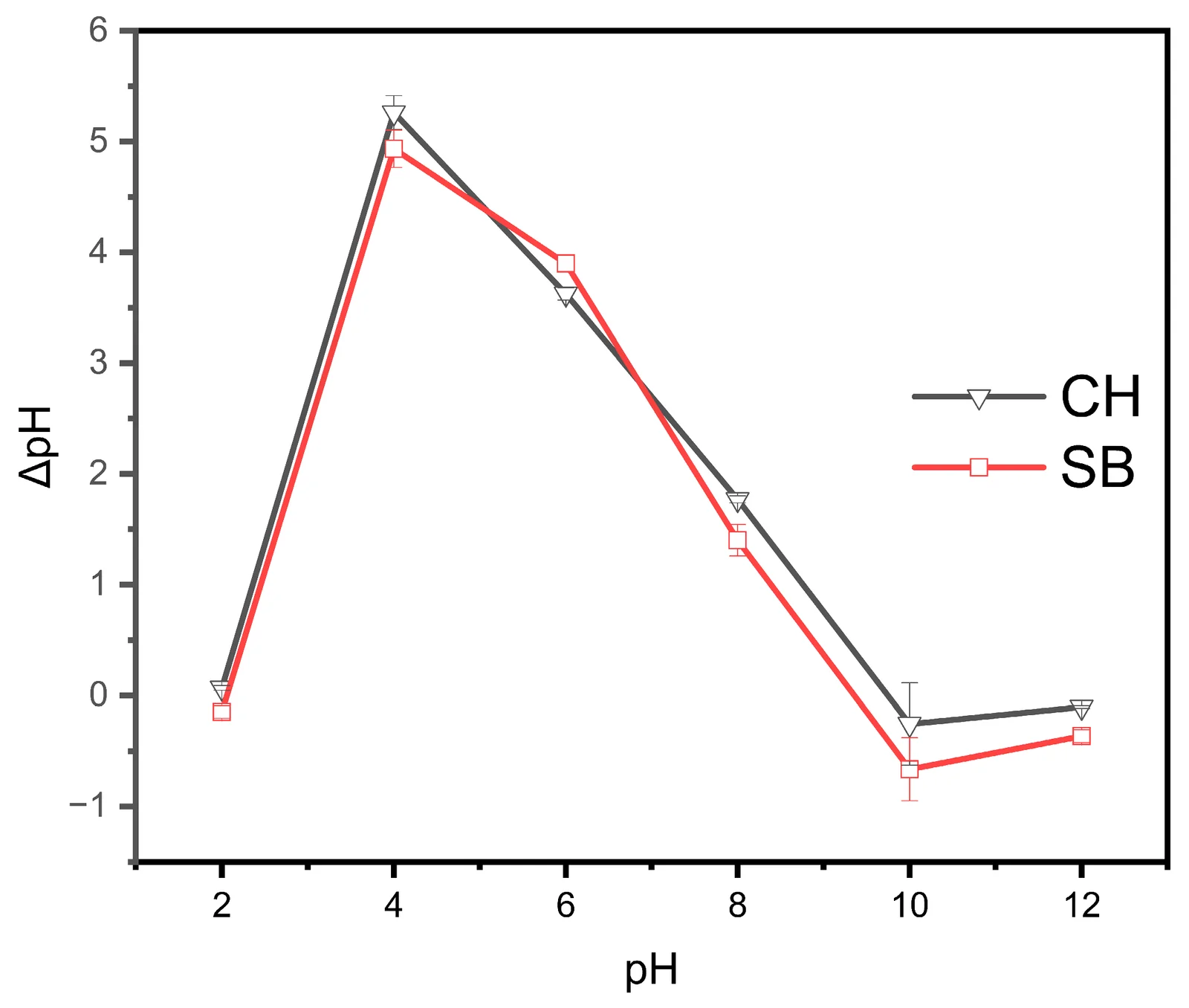
ΔpH variation as a function of initial pH for CH and SB in 0.01 M NaCl solution. The intersection at ΔpH = 0 corresponds to the pH_p_ZC of each material.

**Figure 7:**
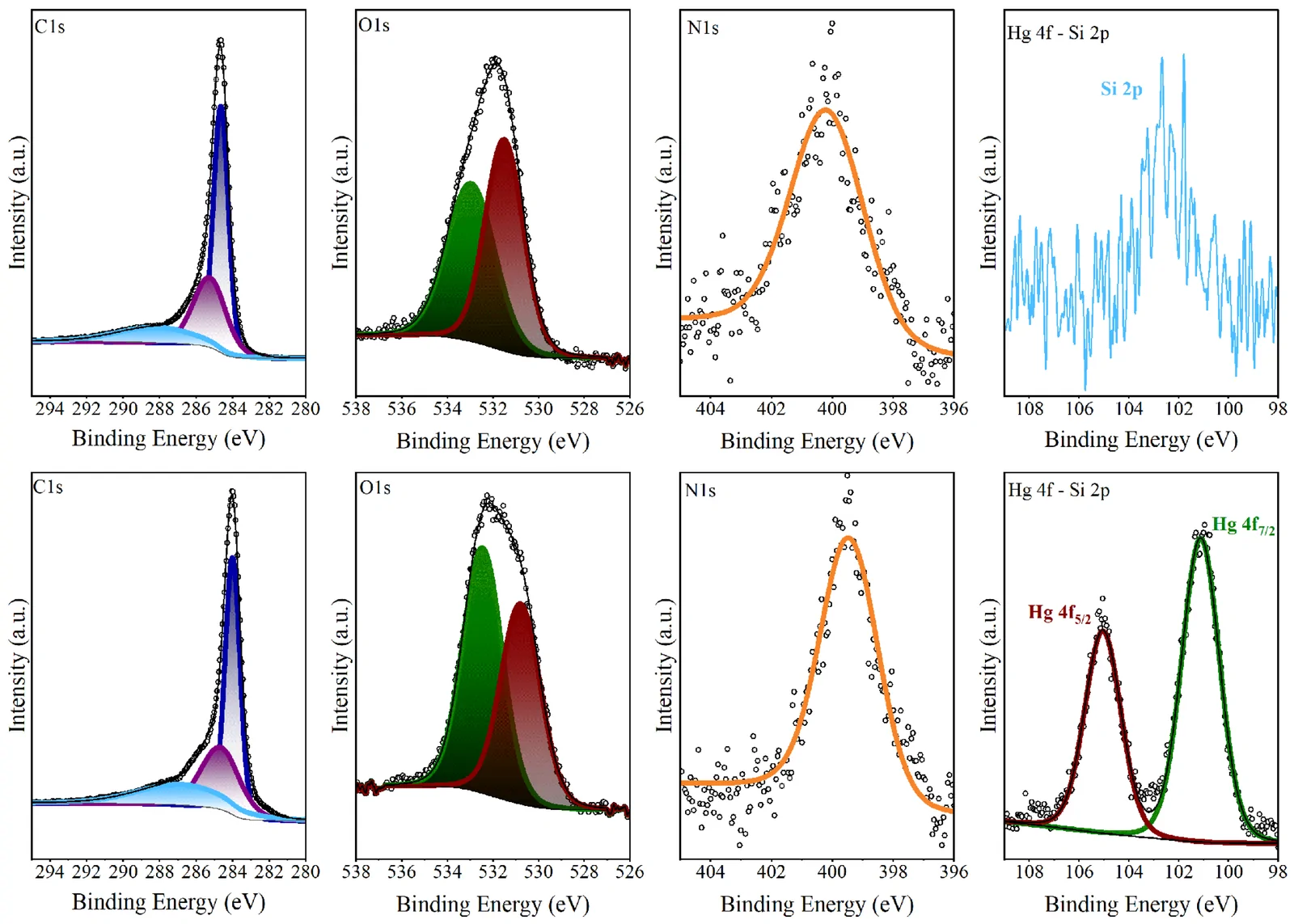
High-resolution XPS spectra of CH: deconvolution of the C1s, O1s, N1s, Si2p, and Hg 4f regions.

**Figure 8:**
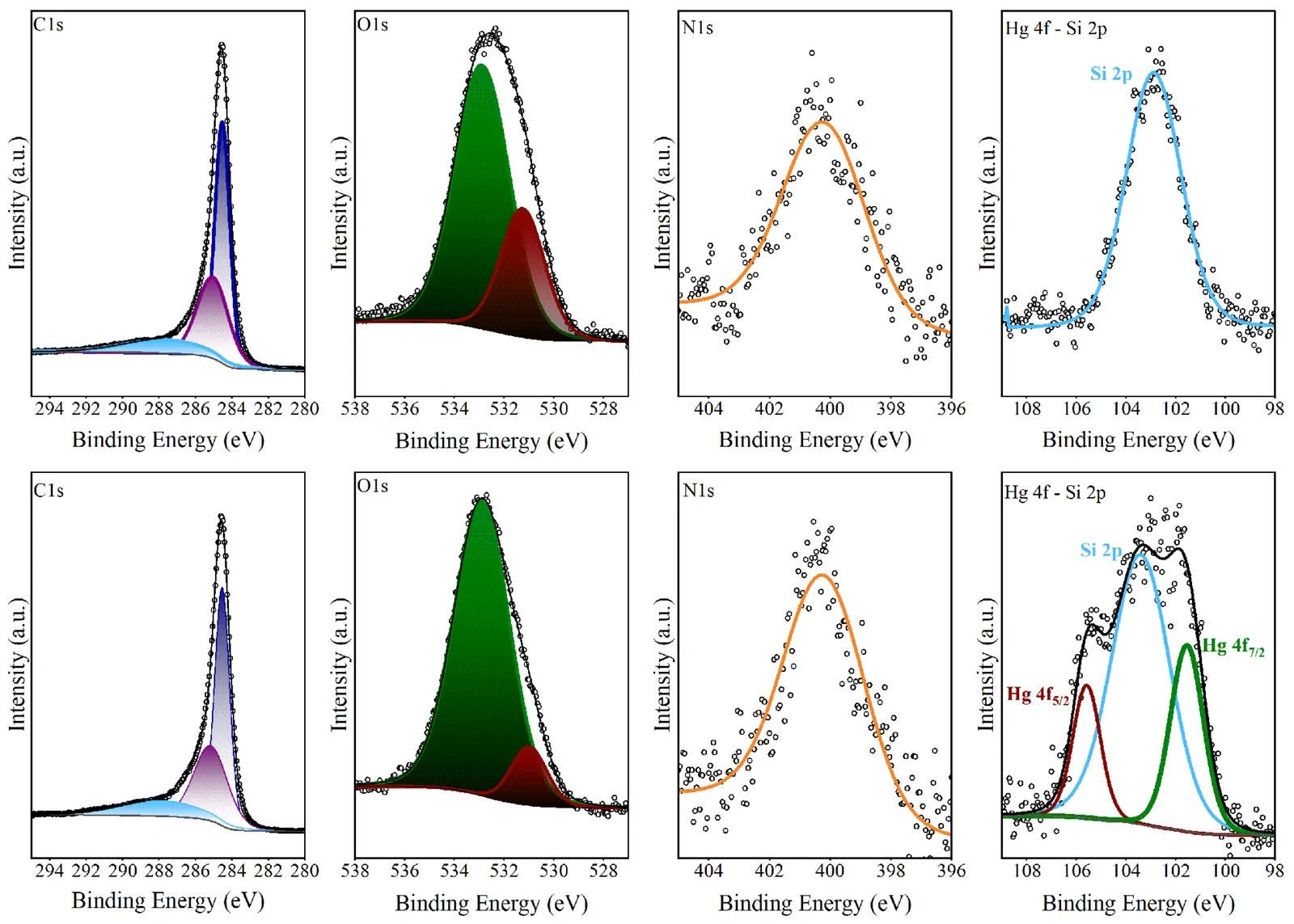
High-resolution XPS spectra of SB: deconvolution of the C1s, O1s, N1s, Si2p, and Hg 4f regions.

**Figure 9:**
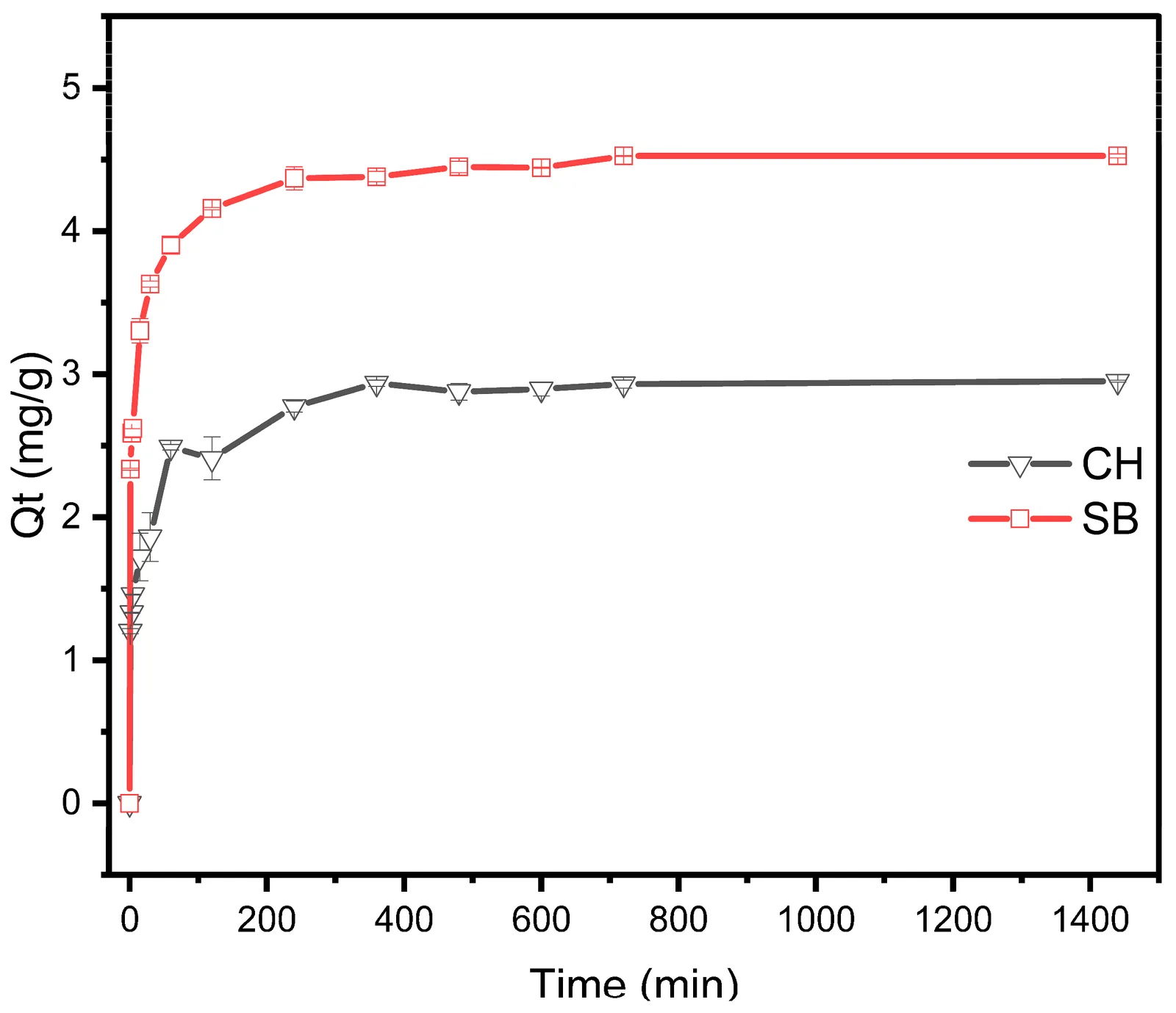
Time-dependent evolution of Hg(II) adsorption capacity (Q_t_) for CH and SB.

**Scheme 1:**
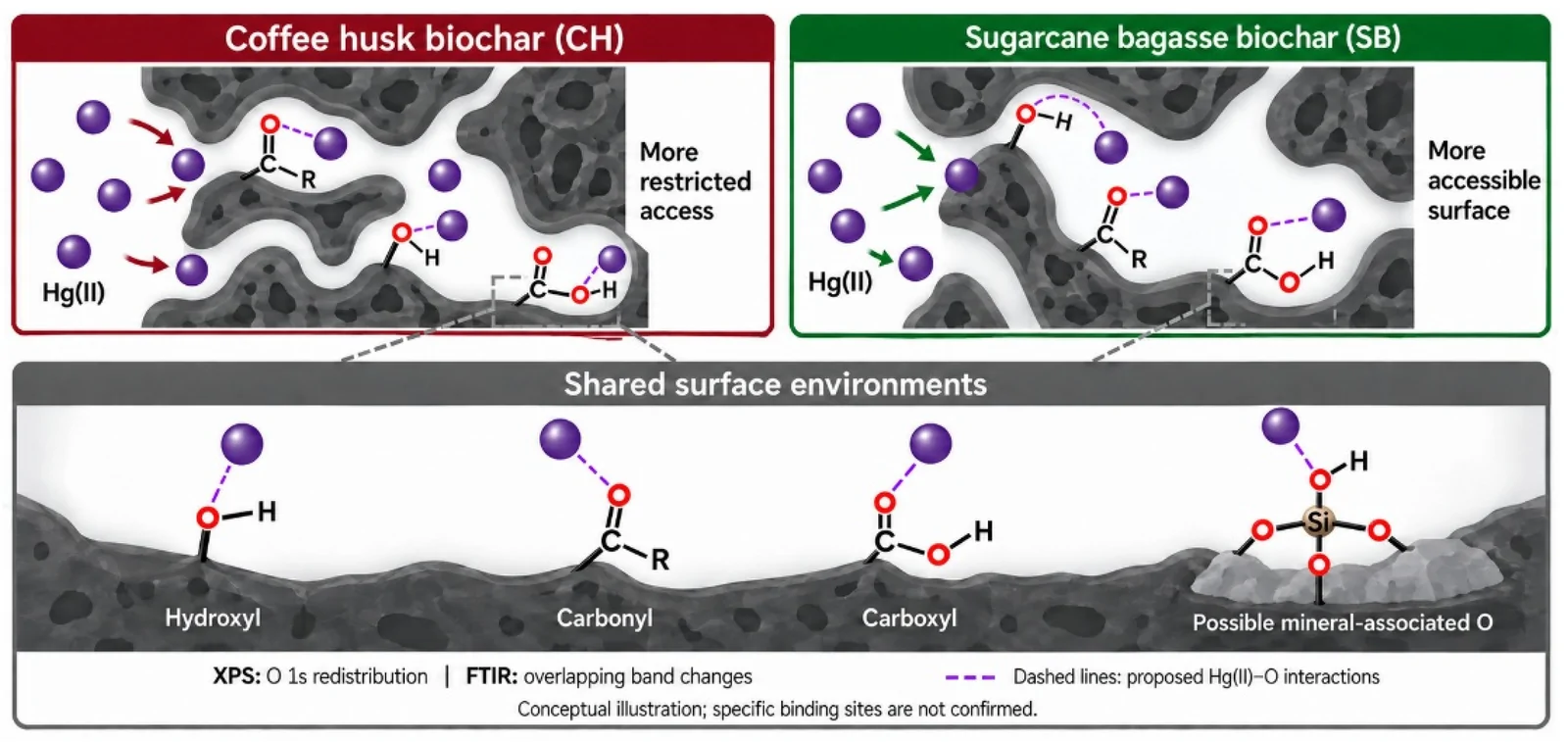
Mechanistic interpretation of Hg(II) adsorption on coffee husk and sugarcane bagasse biochars based on structural, mineralogical, and surface chemical analyses.

**Table 1:** Textural characteristics of CH and SB biochars derived from N_2_ physisorption analysis.

Sample ID	BET Surface Area (m^2^/g)	Total Pore Volume (cm^3^/g)	Micropore Volume (cm^3^/g)	Micropore Fraction (%)	Mean Pore Diameter (Å)
SB	599	0.298	0.189	63.4	19.9
CH	525	0.216	0.186	86.3	16.5

**Table 2:** Elemental composition (wt%) of CH and SB biochars determined by X-ray fluorescence (XRF).

Element	SB (wt%)	CH (wt%)	Element	SB (wt%)	CH (wt%)
K	7.69	8.51	Al	2.27	3.61
Ca	25.80	58.50	Mn	2.66	2.35
P	6.13	2.61	Zn	0.48	1.05
Si	41.20	4.37	Cu	0.17	1.41
Fe	8.28	11.10	Others	2.17	3.79
S	3.15	2.70			

## Data Availability

The original contributions presented in this study are included in the article and Supplementary Materials. Further inquiries can be directed to the corresponding author.

## Supplementary Materials

The following supporting information can be downloaded at: https://www.mdpi.com/article/10.3390/molecules31213914/s1, Figure S1: Kinetic modeling of Hg(II) adsorption on SB biochar using pseudo-first-order, pseudo-second-order, Avrami, Elovich, and Weber–Morris models. Figure S2: Kinetic modeling of Hg(II) adsorption on CH biochar using pseudo-first-order, pseudo-second-order, Avrami, Elovich, and Weber–Morris models. Figure S3: Van’t Hoff plot of ln(Kc) against 1/T for Hg(II) adsorption onto CH biochar. The linear regression was used to estimate the apparent thermodynamic parameters (ΔH° and ΔS°). Figure S4: Van’t Hoff plot of ln(Kc) against 1/T for Hg(II) adsorption onto SB biochar. The linear regression was used to estimate the apparent thermodynamic parameters (ΔH° and ΔS°). Figure S5: Higher-magnification FESEM micrographs of (a) coffee husk biochar (CH, 10,000×) and (b) sugarcane bagasse biochar (SB, 7500×). Table S1: Initial and final pH during the kinetic experiments of Hg(II) adsorption by CH and SB biochars. Table S2: Kinetic parameters for Hg(II) adsorption on CH and SB biochars (0–480 min). Table S3: Freundlich and Langmuir isotherm parameters for Hg(II) adsorption onto SB and CH at different temperatures. The isotherm contact time was 480 min. Table S4: Temperature-dependent apparent equilibrium constants used for the Van’t Hoff analysis of Hg(II) adsorption onto CH and SB. Table S5: Thermodynamic parameters for Hg(II) adsorption onto CH and SB obtained from the Van’t Hoff equation. Text S1: Pseudo-first-order kinetic model. Text S2: Pseudo-second-order kinetic model. Text S3: Avrami kinetic model. Text S4: Elovich kinetic model. Text S5: Weber–Morris intraparticle diffusion model. Text S6: Langmuir adsorption isotherm. Text S7: Freundlich adsorption isotherm. Text S8: Thermodynamic parameters. References [24,97,98,99,100,101,102,103,104,105,106,107,108,109] are also cited in the Supplementary Materials.

## Abbreviations

The following abbreviations are used in this manuscript:

Abbreviation	Definition
BET	Brunauer–Emmett–Teller
CH	Coffee Husk Perisperm (Silver Skin) Biochar
SB	Sugarcane Bagasse Biochar
FESEM	Field Emission Scanning Electron Microscopy
EDS	Energy-Dispersive X-ray Spectroscopy
SEM	Scanning Electron Microscopy
FTIR	Fourier-Transform Infrared Spectroscopy
XPS	X-ray Photoelectron Spectroscopy
XRD	X-ray Diffraction
XRF	X-ray Fluorescence
PFO	Pseudo-First-Order Model
PSO	Pseudo-Second-Order Model
PDF	Powder Diffraction File
IUPAC	International Union of Pure and Applied Chemistry
STP	Standard Temperature and Pressure
Hg(II)	Divalent Mercury
SBET	Specific Surface Area Determined by BET Method
RA-915	Mercury Analyzer RA-915
