# Supplementary material for: Comparative Hg(II) Adsorption on Coffee Husk and Sugarcane Bagasse Biochars: Structure–Function Relationships
Source: Molecules. 2026 Oct 30;31(21):3914. doi: 10.3390/molecules31213914 (PMC13765511; doi:10.3390/molecules31213914)
Supplement: Supplementary file 1 [file molecules-31-03914-s001.zip › molecules-4632696-supplementary.pdf]

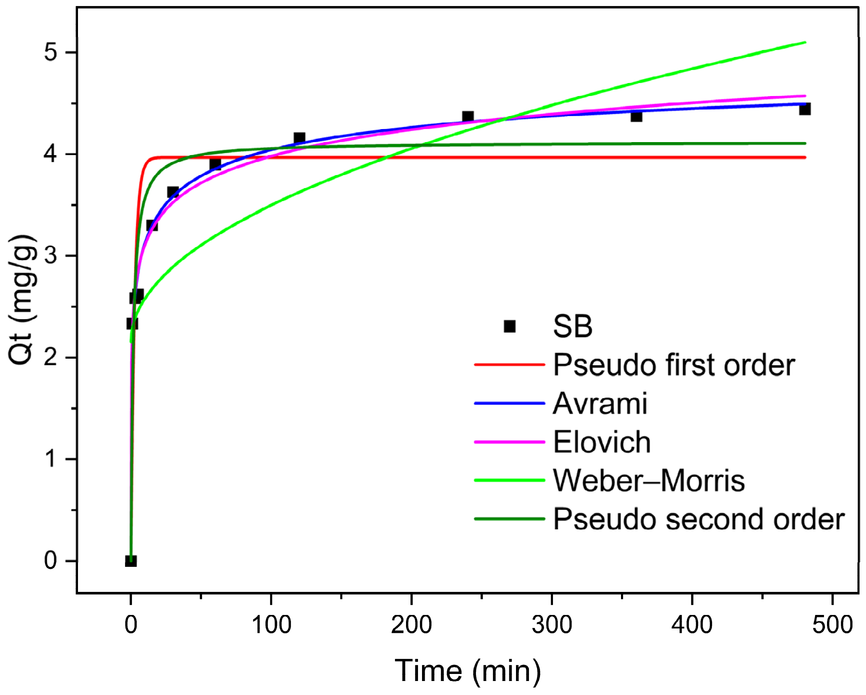

Figure S1. Kinetic modeling of Hg (II) adsorption on SB biochar using pseudo-first-order, pseudo-second-order, Avrami, Elovich, and Weber–Morris models.

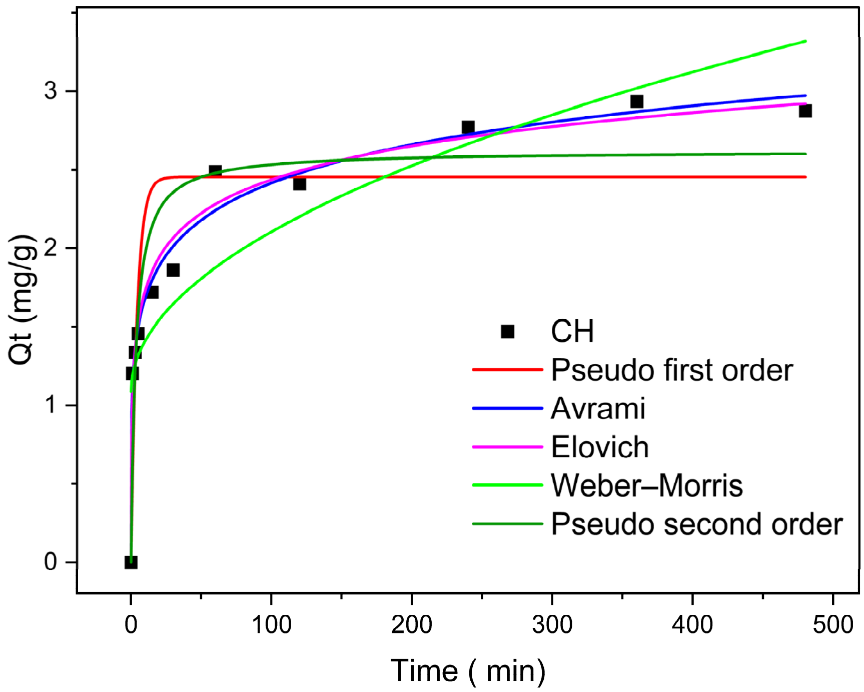

Figure S2. Kinetic modeling of Hg(II) adsorption on CH biochar using pseudo-first order, pseudo-second order, Avrami, Elovich and Weber-Morris models.

Table S1. Initial and final pH during the kinetic experiments of Hg(II) adsorption by CH and SB biochars

Biochar	Initial pH	Final pH
**CH**	6.58 ± 0.09	7.62 ± 0.01
**SB**	6.53 ± 0.11	7.19 ± 0.03

Table S2. Kinetic parameters for Hg(II) adsorption on CH and SB biochars (0–480 min).

**Model**	**Parameter**	**SB**	**CH**
**Pseudo-First-Order**	qe (mg g^{-1})	4.06 ± 0.20	2.45 ± 0.18
	k_1 (min^{-1})	0.36 ± 0.10	0.237 ± 0.086
	Reduced χ^2	0.326	0.205
	R^2	0.831	0.771
	Adj. R^2	0.814	0.745
**Pseudo-Second-Order**	qe (mg g^{-1})	4.20 ± 0.15	2.62 ± 0.15
	k_2 (g mg^{-1} min^{-1})	0.140 ± 0.042	0.115 ± 0.045
	Reduced χ^2	0.159	0.117
	R^2	0.918	0.869
	Adj. R^2	0.910	0.855
**Avrami**	qe (mg g^{-1})	4.78 ± 0.15	4.5 ± 2.3
	kAV (min^{-1})	0.129 ± 0.034	0.003 ± 0.013
	nAV	0.244 ± 0.021	0.218 ± 0.061
	Reduced χ^2	0.0095	0.0152
	R^2	0.996	0.985
	Adj. R^2	0.995	0.981
**Elovich**	α	230 ± 130	8.4 ± 3.7
	β	2.82 ± 0.16	3.24 ± 0.23
	Reduced χ^2	0.0216	0.018
	R^2	0.989	0.980
	Adj. R^2	0.988	0.978
**Weber–Morris**	kid (mg g^{-1} $min^{-1}/^2$)	0.083 ± 0.026	0.102 ± 0.019
	C	2.48 ± 0.40	1.09 ± 0.21
	Reduced χ^2	0.96	0.216
	R^2	0.503	0.758
	Adj. R^2	0.453	0.731

Table S3. Freundlich and Langmuir isotherm parameters for Hg(II) adsorption onto SB and CH at different temperatures. The isotherm contact time was 480 min.

**Material**	**T (K)**	**Freundlich R^2**	**1/n**	**Intercept**	$\boldsymbol{K_F}$	**Langmuir R^2**	**Slope**	**Intercept**	$\boldsymbol{q_m}$ **(mg g^{-1})**	$\boldsymbol{K_L}$
**SB**	293.15	0.970	0.333	2.19	8.92	0.950	0.0478	0.0418	20.92	1.14
	298.15	0.957	0.388	2.19	8.94	0.980	0.0405	0.0513	24.69	0.790
	308.15	0.981	0.553	1.52	4.57	0.995	0.0387	0.182	25.84	0.213
	313.15	0.964	0.597	1.65	5.23	0.985	0.0298	0.170	33.56	0.175
	318.15	0.995	0.652	1.66	5.24	0.975	0.0222	0.172	45.05	0.129
**CH**	293.15	0.985	0.334	1.44	4.20	0.975	0.0796	0.171	12.56	0.465
	298.15	0.977	0.403	1.42	4.15	0.989	0.0713	0.182	14.03	0.392
	308.15	0.984	0.623	1.27	3.55	0.967	0.0370	0.253	27.03	0.146
	313.15	0.909	0.671	1.28	3.59	0.871	0.0296	0.202	33.78	0.146
	318.15	0.977	0.739	1.27	3.55	0.962	0.0221	0.259	45.25	0.0854

Table S4. Temperature-dependent apparent equilibrium constants used for the Van't Hoff analysis of Hg(II) adsorption onto CH and SB.

Material	***T (K)***	***1/T (K^{-1})***	***Kc***	***Ln(Kc)***
CH	293.15	3.411×10^{-3}	5.13×10^{6}	15.450
CH	298.15	3.354×10^{-3}	4.37×10^{6}	15.290
CH	308.15	3.245×10^{-3}	1.63×10^{6}	14.304
CH	313.15	3.193×10^{-3}	1.62×10^{6}	14.296
CH	318.15	3.143×10^{-3}	9.51×10^{5}	13.765
SB	293.15	3.411×10^{-3}	1.25×10^{7}	16.345
SB	298.15	3.354×10^{-3}	8.79×10^{6}	15.989
SB	308.15	3.245×10^{-3}	2.37×10^{6}	14.677
SB	313.15	3.193×10^{-3}	1.92×10^{6}	14.470
SB	318.15	3.143×10^{-3}	1.44×10^{6}	14.181

Table S5. Thermodynamic parameters for Hg(II) adsorption onto CH and SB obtained from the Van't Hoff equation.

Material	Slope (m)	Intercept (b)	ΔH° (kJ mol^{-1})	ΔS° (J mol^{-1} K^{-1})	$ΔG°_{(298)}$ (kJ mol^{-1})
**CH**	6350	−6.125	−52.8	−50.9	−37.6
**SB**	8590	−12.956	−71.4	−107.7	−39.3

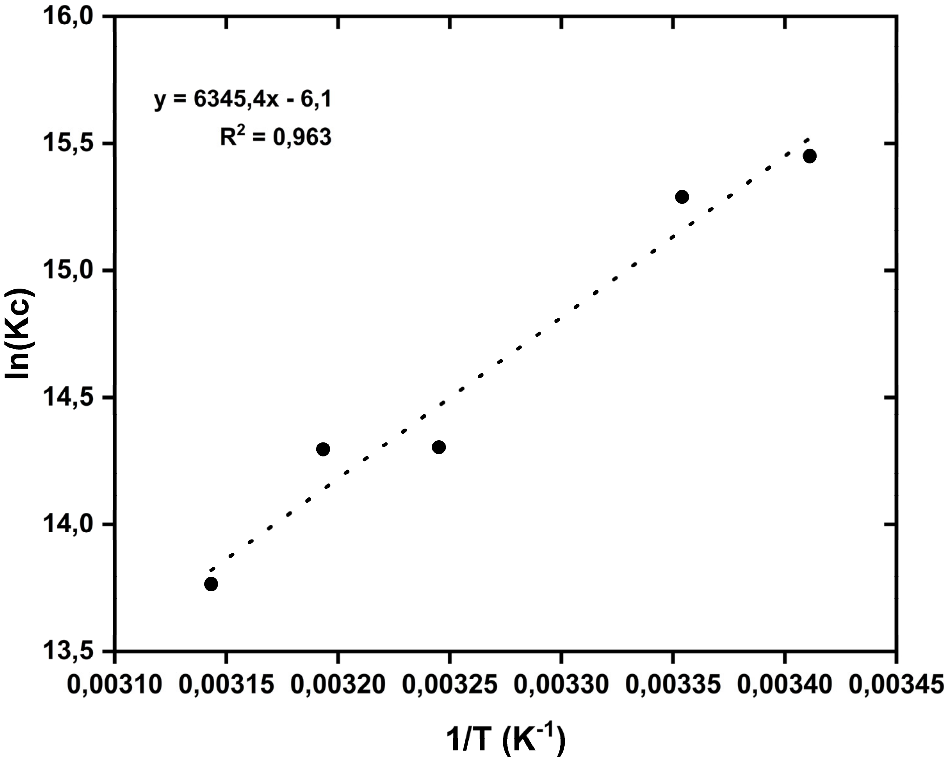

Figure S3. Van't Hoff plot of ln(Kc) against 1/T for Hg(II) adsorption onto CH biochar. The linear regression was used to estimate the apparent thermodynamic parameters (ΔH° and ΔS°).

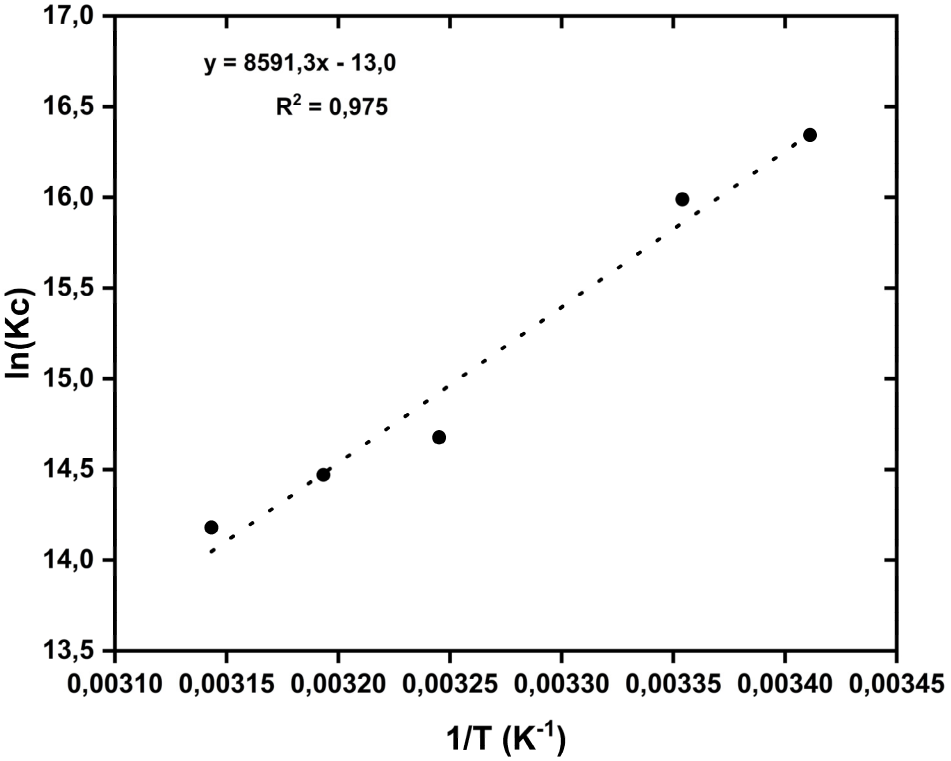

Figure S4. Van’t Hoff plot of ln(Kc) against 1/T for Hg(II) adsorption onto SB biochar. The linear regression was used to estimate the apparent thermodynamic parameters (ΔH° and ΔS°).

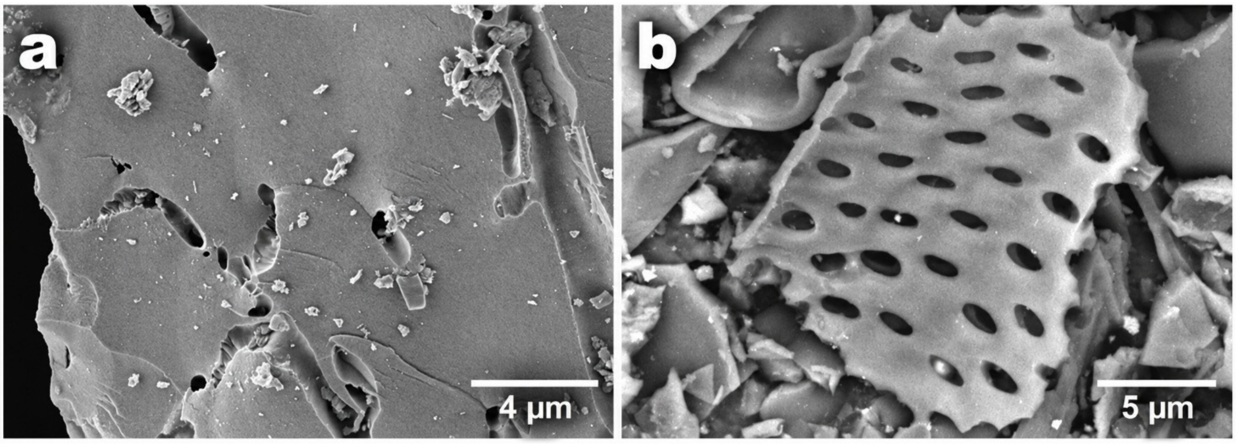

Figure S5. Higher-magnification FESEM micrographs of (a) coffee husk biochar (CH, 10,000×) and (b) sugarcane bagasse biochar (SB, 7,500×)

Text S1 Pseudo first order kinetic model

The pseudo-first-order (PFO) model describes the evolution of adsorption capacity with contact time using the following nonlinear expression [1]:

$$q_t = q_e[1 - \exp(-k_1 t)] \qquad \text{(S1)}$$

Here, q_t and q_e are the adsorption capacities at time t and at equilibrium (mg g^{-1}), respectively; t is the contact time (min), and k_1 is the PFO rate constant (min^{-1}). The experimental equilibrium capacity is calculated by mass balance:

$$q_e = \frac{(C_0 - C_e)V}{m} \qquad \text{(S2)}$$

C_0 and C_e are the initial and equilibrium aqueous Hg(II) concentrations (mg L^{-1}), respectively; V is the solution volume (L), and m is the dry mass of adsorbent (g).

The conventional linearized PFO expression is:

$$\ln(q_e - q_t) = \ln q_e - k_1 t \qquad \text{(S3)}$$

A plot of $\ln(q_e - q_t)$ against t has slope $-k_1$ and intercept $\ln(q_e)$. The transformation requires $q_e > q_t$ and an independently specified or iteratively estimated q_e; points at or above q_e cannot be included in this logarithmic plot.

Text S2 Pseudo second order kinetic model

The pseudo-second-order (PSO) model is expressed in nonlinear form as [2]:

$$q_t = \frac{k_2 {q_e}^2 t}{1 + k_2 q_e t} \qquad \text{(S4)}$$

The PSO rate constant k_2 has units of g mg^{-1} min^{-1}. The definitions of q_t, q_e, and t are the same as in Text S1. A good fit to this equation does not, by itself, establish a chemisorption mechanism.

A commonly used linearized PSO expression is:

$$\frac{t}{q_t} = \frac{1}{k_2 {q_e}^2} + \frac{t}{q_e} \qquad \text{(S5)}$$

For a plot of t/q_t against t, the slope s equals $1/q_e$ and the intercept i equals $1/(k_2 q_e^2)$. Thus, $q_e = 1/s$ and $k_2 = s^2/i$. The undefined point $t = 0$, $q_t = 0$ is excluded.

Text S3 Avrami kinetic model

The Avrami model provides an empirical description of adsorption kinetics with a time dependence controlled by an additional exponent [3]:

$$q_t = q_e[1 - \exp(-(k_{AV} t)^{n_{AV}})] \qquad \text{(S6)}$$

For this parametrization, k_{AV} is the Avrami rate constant (min^{-1}), and n_{AV} is dimensionless. The exponent allows deviations from a single exponential uptake curve. It should not be interpreted as direct evidence of a specific molecular adsorption mechanism.

Taking natural logarithms twice gives the linearized form:

$$\ln[-\ln(1 - \frac{q_t}{q_e})] = n_{AV} \ln k_{AV} + n_{AV} \ln t \qquad \text{(S7)}$$

A plot of $\ln[-\ln(1 - q_t/q_e)]$ against $\ln(t)$ has slope $s = n_{AV}$ and intercept $i = n_{AV} \ln(k_{AV})$, giving $k_{AV} = \exp(i/s)$. This transformation requires $t > 0$ and $0 < q_t/q_e < 1$, as well as a specified or

independently estimated q_e. The equation corresponds specifically to the parametrization in which k_{AV} has units of min^{-1}.

Text S4 Elovich kinetic model

The Elovich equation describes an adsorption rate that decreases exponentially as the adsorbed amount increases [4]:

$$\frac{dq_t}{dt} = \alpha \exp(-\beta q_t) \qquad \text{(S8)}$$

Integration with the initial condition $q_t = 0$ at $t = 0$ gives:

$$q_t = \frac{\ln(1+\alpha\beta t)}{\beta} \qquad \text{(S9)}$$

Here, α is the initial adsorption rate (mg g^{-1} min^{-1}), and β is the Elovich coefficient (g mg^{-1}), which describes how rapidly the rate decreases with increasing adsorption capacity. When αβt ≫ 1, the commonly used approximation is:

$$q_t \approx \frac{\ln(\alpha\beta)}{\beta} + \frac{\ln t}{\beta} \qquad \text{(S10)}$$

The approximate expression applies for t > 0 and should be used only when its limiting condition is satisfied. Although this model is often used for adsorption on heterogeneous surfaces, its fit alone does not identify the binding mechanism.

This approximation is the conventional linearized Elovich form. In a plot of q_t against ln(t), the slope s equals 1/β and the intercept i equals ln(αβ)/β. Consequently, β = 1/s and α = s exp(i/s), with t entered in minutes. It is not an exact linearization of the full integrated equation when αβt is not much greater than one.

Text S5 Weber Morris intraparticle diffusion model

The Weber–Morris relationship is used to examine the contribution of intraparticle diffusion to the adsorption time profile [5]:

$$q_t = k_{id} t^{\frac{1}{2}} + C \qquad \text{(S11)}$$

The coefficient k_id is the intraparticle diffusion rate constant (mg g^{-1} $min^{-1}/^{2}$), and C is the intercept (mg g^{-1}). C reflects the contribution of processes not represented by the diffusion term and is often discussed in relation to boundary-layer effects; it is not a direct measurement of boundary-layer thickness. A single linear relationship passing through the origin is consistent with intraparticle diffusion as the sole rate-controlling contribution within this model. A nonzero intercept or multiple linear regions indicates that this description alone is insufficient to represent the full uptake profile.

The equation is already linear in $t^{1/2}$: plotting q_t against $t^{1/2}$ yields k_id as the slope and C as the intercept. If distinct linear regions are fitted, their time intervals and corresponding slopes and intercepts should be reported separately.

Text S6 Langmuir adsorption isotherm

The Langmuir isotherm describes adsorption onto an idealized population of equivalent sites with a finite monolayer capacity [6]:

$$q_e = \frac{q_m K_L C_e}{1+K_L C_e} \qquad \text{(S12)}$$

Here, q_m is the model-estimated maximum adsorption capacity (mg g^{-1}), K_L is the Langmuir affinity constant (L mg^{-1}), and C_e is the equilibrium concentration (mg L^{-1}). Capacity and affinity are distinct model parameters: a larger q_m does not necessarily imply a larger K_L. The dimensionless separation factor can be calculated as [7]:

$$R_L = \frac{1}{1+K_L C_0} \quad \text{(S13)}$$

For a specified initial concentration C_0, $0 < R_L < 1$ conventionally denotes favorable adsorption; $R_L = 1$ corresponds to a linear limiting case, $R_L > 1$ to unfavorable adsorption, and $R_L \rightarrow 0$ to the limiting case conventionally described as irreversible.

The commonly used concentration-over-capacity linearization is:

$$\frac{C_e}{q_e} = \frac{1}{q_m K_L} + \frac{C_e}{q_m} \text{(S14)}$$

A plot of C_e/q_e against C_e has slope $s = 1/q_m$ and intercept $i = 1/(q_m K_L)$; therefore, $q_m = 1/s$ and $K_L = s/i$. Only points with $q_e > 0$ are included.

Text S7 Freundlich adsorption isotherm

The Freundlich isotherm is an empirical power-law expression commonly used to describe adsorption on energetically heterogeneous surfaces [8–10]:

$$q_e = K_F C_e^{\frac{1}{n}} \quad \text{(S15)}$$

K_F is the Freundlich coefficient, with units of (mg g^{-1})(L mg^{-1})$^{1/n}$ when q_e and C_e are expressed in mg g^{-1} and mg L^{-1}, respectively. The dimensionless exponent 1/n describes the curvature of the isotherm; $0 < 1/n < 1$ is commonly associated with favorable adsorption over the fitted concentration range. Unlike the Langmuir model, the Freundlich equation does not predict a finite saturation capacity. Its parameters should therefore be interpreted within the experimental concentration range.

The logarithmic linearization is:

$$\ln q_e = \ln K_F + \frac{1}{n} \ln C_e \quad \text{(S16)}$$

For positive q_e and C_e values, a plot of $\ln(q_e)$ against $\ln(C_e)$ has slope $s = 1/n$ and intercept $i = \ln(K_F)$. Hence, $n = 1/s$ and $K_F = \exp(i)$, using the stated units for capacity and concentration.

Text S8 Thermodynamic parameters

The apparent standard enthalpy and entropy changes are estimated from the temperature dependence of a dimensionless equilibrium constant using the van't Hoff relationship [11–13]:

$$\ln K_c = \frac{\Delta S^{\circ}}{R} - \frac{\Delta H^{\circ}}{RT} \quad \text{(S17)}$$

$$\Delta G^{\circ} = \Delta H^{\circ} - T\Delta S^{\circ} = -RT \ln K_c \quad \text{(S18)}$$

$\Delta G°$, $\Delta H°$, and $\Delta S°$ denote the apparent standard Gibbs energy, enthalpy, and entropy changes, respectively; T is the absolute temperature (K), and R is the gas constant (8.314 J mol^{-1} K^{-1}). For a linear plot of $\ln(K_c)$ against 1/T, the slope equals $-\Delta H°/R$ and the intercept equals $\Delta S°/R$. This treatment assumes that $\Delta H°$ and $\Delta S°$ are approximately constant over the investigated temperature interval.

Following the conversion convention reported in the adsorption literature [12,14], the Langmuir constant was converted into a dimensionless apparent equilibrium constant as follows:

$$K_c = \mathrm{MW} \times 55.5 \times 1000 \times K_L \text{ (S19)}$$

$$K_c = K_L\ [\mathrm{L\ mg^{-1}}] \times 1000\ [\mathrm{mg\ g^{-1}}] \times M_{Hg}\ [\mathrm{g\ mol^{-1}}] \times 55.5\ [\mathrm{mol\ L^{-1}}]$$

Here, MW is the molar mass of Hg (200.59 g mol^{-1}), K_l is expressed in L mg^{-1}, and 1000 mg g^{-1} converts the mass units. The factor 55.5 mol L^{-1} is the approximate molar concentration of water and defines the adopted dilute-solution mole-fraction convention, x ≈ c/(55.5 mol L^{-1}). Under the ideal-dilute Langmuir approximation, all units cancel and Kc is dimensionless. The same numerical factor 55.5 was used at all investigated temperatures.

Relative to a 1 mol L^{-1} concentration standard, the adopted dimensionless constant is multiplied by 55.5. Adding the temperature-independent term ln(55.5) changes the van't Hoff intercept but not its slope. Thus, ΔH° is unchanged; ΔS° increases by R ln(55.5) ≈ 33.4 J mol^{-1} K^{-1}, and ΔG° decreases by RT ln(55.5) ≈ 9.96 kJ mol^{-1} at 298.15 K. The negative apparent enthalpy is therefore not an artifact of including this constant factor. The absolute values of ΔG° and ΔS° remain conditional on the adopted standard state.

With R expressed in J mol^{-1} K^{-1}, ΔH° and ΔG° are obtained in J mol^{-1} and may be divided by 1000 for reporting in kJ mol^{-1}; ΔS° is expressed in J mol^{-1} K^{-1}. Negative ΔG° values indicate thermodynamic favorability under the adopted standard-state convention, while the sign of ΔH° distinguishes exothermic from endothermic behavior.

References

1. Chu, K.H.; Bollinger, J.-C.; Kierczak, J. Pseudo-first-order kinetics in environmental adsorption: Why are there two distinct equations? *Environ. Surf. Interfaces* **2025**, *3*, 191–195. https://doi.org/10.1016/j.esi.2025.07.001.

2. Ho, Y.S.; McKay, G. Pseudo-second order model for sorption processes. *Process Biochem.* **1999**, *34*, 451–465. https://doi.org/10.1016/S0032-9592(98)00112-5.

3. Lopes, E.C.N.; dos Anjos, F.S.C.; Vieira, E.F.S.; Cestari, A.R. An alternative Avrami equation to evaluate kinetic parameters of the interaction of Hg(II) with thin chitosan membranes. *J. Colloid Interface Sci.* **2003**, *263*, 542–547. https://doi.org/10.1016/S0021-9797(03)00326-6.

4. Chien, S.H.; Clayton, W.R. Application of Elovich equation to the kinetics of phosphate release and sorption in soils. *Soil Sci. Soc. Am. J.* **1980**, *44*, 265–268. https://doi.org/10.2136/sssaj1980.03615995004400020013x.

5. Weber, W.J., Jr.; Morris, J.C. Kinetics of adsorption on carbon from solution. *J. Sanit. Eng. Div.* **1963**, *89*, 31–59. https://doi.org/10.1061/JSEDAI.0000430.

6. Sun, Z.; Tian, C.; Yang, T.; Fu, J.; Xu, H.; Wang, Y.; Lin, Z. A MOF-based trap with strong affinity toward low-concentration heavy metal ions. *Sep. Purif. Technol.* **2022**, *301*, 121946. https://doi.org/10.1016/j.seppur.2022.121946.

7. Imla Syafiqah, M.S.; Yussof, H.W. Kinetics, isotherms, and thermodynamic studies on the adsorption of mercury (II) ion from aqueous solution using modified palm oil fuel ash. *Mater. Today Proc.* **2018**, *5*, 21690–21697. https://doi.org/10.1016/j.matpr.2018.07.020.

8. Nasir, M.Z.M.; Zaini, M.A.A.; Yunus, M.A.C. Adsorption profiles of rhodamine B and reactive orange 16 onto pharmaceutical-based activated charcoals. *Desalin. Water Treat.* **2018**, *132*, 340–349. https://doi.org/10.5004/dwt.2018.23152.

9. Chiban, M.; Soudani, A.; Sinan, F.; Persin, M. Single, binary and multi-component adsorption of some anions and heavy metals on environmentally friendly *Carpobrotus edulis* plant. *Colloids Surf. B Biointerfaces* **2011**, *82*, 267–276. https://doi.org/10.1016/j.colsurfb.2010.09.013.

10. Dada, A.O.; Ojediran, J.O.; Olalekan, A.P. Sorption of Pb^{2+} from aqueous solution unto modified rice husk: Isotherms studies. *Adv. Phys. Chem.* **2013**, *2013*, 842425. https://doi.org/10.1155/2013/842425.

11. Medici, F.; Patterer, M.S.; Peluso, M.A.; Sambeth, J.E. Lead adsorption from aqueous solution using manganese oxides recovered from spent alkaline batteries. *J. Solid Waste Technol. Manag.* **2020**, *46*, 206–212. https://doi.org/10.5276/JSWTM/2020.206.

12. Paredes-Laverde, M.; Silva-Agredo, J.; Torres-Palma, R.A. Removal of norfloxacin in deionized, municipal water and urine using rice (*Oryza sativa*) and coffee (*Coffea arabica*) husk wastes as natural adsorbents. *J. Environ. Manage.* **2018**, *213*, 98–108. https://doi.org/10.1016/j.jenvman.2018.02.047.

13. Cantu, Y.; Remes, A.; Reyna, A.; Martinez, D.; Villarreal, J.; Ramos, H.; Trevino, S.; Tamez, C.; Martinez, A.; Eubanks, T.; Parsons, J.G. Thermodynamics, kinetics, and activation energy studies of the sorption of chromium(III) and chromium(VI) to a Mn_3O_4 nanomaterial. *Chem. Eng. J.* **2014**, *254*, 374–383. https://doi.org/10.1016/j.cej.2014.05.110

14. Zhou, X.; Zhou, X. The Unit Problem in the Thermodynamic Calculation of Adsorption Using the Langmuir Equation. Chemical Engineering Communications 2014, 201, 1459–1467. DOI: 10.1080/00986445.2013.818541.
